# The *MKK7* p.Glu116Lys Rare Variant Serves as a Predictor for Lung Cancer Risk and Prognosis in Chinese

**DOI:** 10.1371/journal.pgen.1005955

**Published:** 2016-03-30

**Authors:** Fuman Qiu, Lei Yang, Xiaoxiao Lu, Jiansong Chen, Di Wu, Yongfang Wei, Qingqing Nong, Lisha Zhang, Wenxiang Fang, Xiaoliang Chen, Xiaoxuan Ling, Binyao Yang, Xin Zhang, Yifeng Zhou, Jiachun Lu

**Affiliations:** 1 The State Key Lab of Respiratory Disease, The Institute for Chemical Carcinogenesis, Collaborative Innovation Center for Environmental Toxicity, School of Public Health, Guangzhou Medical University, Guangzhou, People's Republic of China; 2 Biomedicine Research Center and Department of Surgery, The Third Affiliated Hospital of Guangzhou Medicine University, Guangzhou, People's Republic of China; 3 School of Arts and Sciences, Colby-Sawyer College, New London, New Hampshire, United States of America; 4 Center of Laboratory Animal, Guangzhou Medical University, Guangzhou, People's Republic of China; 5 Department of Environmental Health, Guangxi Medical University, Nanning, People's Republic of China; 6 Department of Thoracic Surgery, The First Affiliated Hospital of Guangzhou Medical University, Guangzhou, People's Republic of China; 7 Department of Genetics, Medical College of Soochow University, Suzhou, People's Republic of China; Dana Farber Cancer Institute, UNITED STATES

## Abstract

Accumulated evidence indicates that rare variants exert a vital role on predisposition and progression of human diseases, which provides neoteric insights into disease etiology. In the current study, based on three independently retrospective studies of 5,016 lung cancer patients and 5,181 controls, we analyzed the associations between five rare polymorphisms (i.e., p.Glu116Lys, p.Asn118Ser, p.Arg138Cys, p.Ala195Thr and p.Leu259Phe) in *MKK7* and lung cancer risk and prognosis. To decipher the precise mechanisms of *MKK7* rare variants on lung cancer, a series of biological experiments was further performed. We found that the *MKK7* p.Glu116Lys rare polymorphism was significantly associated with lung cancer risk, progression and prognosis. Compared with Glu/Glu common genotype, the 116Lys rare variants (Lys/Glu/+ Lys/Lys) presented an adverse effect on lung cancer susceptibility (odds ratio [OR] = 3.29, 95% confidence interval [CI] = 2.70–4.01). These rare variants strengthened patients’ clinical progression that patients with 116Lys variants had a significantly higher metastasis rate and advanced N, M stages at diagnosis. In addition, the patients with 116Lys variants also contributed to worse cancer prognosis than those carriers with Glu/Glu genotype (hazard ratio [HR] = 1.53, 95% CI = 1.32–1.78). Functional experiments further verified that the *MKK7* p.116Lys variants altered the expression of several cancer-related genes and thus affected lung cancer cells proliferation, tumor growth and metastasis *in vivo* and *in vitro*. Taken together, our findings proposed that the *MKK7* p.Glu116Lys rare polymorphism incurred a pernicious impact on lung cancer risk and prognosis through modulating expressions of a serial of cancer-related genes.

## Author Summary

Rare variants have been identified to be associated with a variety of human malignancies, which account for a considerable fraction of heredity for complex diseases. To date, however, the precise molecular mechanism of rare variants involved in tumors initiation and progression largely remains unclear. We tested the associations between rare variants in *MKK7* and lung cancer risk and prognosis in two-stage retrospective studies with a total of 5,016 lung cancer patients and 5,181 controls in Chinese. We found that the rare variant from Glu to Lys in *MKK7* p.116 locus exerted a detrimental effect on lung cancer risk, progression and prognosis. Further functional experiments demonstrated that lung cancer cells with p.116Lys variant accelerated the potentials of cell growth, proliferation, colony formation, migration and invasion than the cells with p.116Glu. This rare variant also promoted the xenograft growth and metastasis of nude mice *in vivo* through regulating a serial of cancer-related genes. Our data indicated that p.Glu116Lys rare variant in *MKK7* might be a novel biomarker for lung cancer risk and prognosis.

## Introduction

Ever-increasing epidemiological studies, especially the genome-wide association studies (GWAS), have extensively identified numerous genetic variants, including single-nucleotide polymorphisms (SNPs), to be associated with risk and progression of various human malignancies[1–3]. Despite these discoveries, much of the genetic contributions to complex diseases remains unclearly illuminated because of the fact that only a small proportion of cancer heritability can be explained by those common SNPs, typically with minor allele frequency (MAF) >5%, reflecting that some ‘missing heritability’ existed [4, 5]. Recently, accumulating evidence revealed that rare variants (MAF<1%) could decipher accessional disease risk or trait variability [6–8]. An example is that the rare variants located in proto-oncogenes or tumor suppressor genes may contribute to phenotypic variations through modifying their biological functions or genes expression, and thus play an important role in cancer initiations and progressions[9, 10]. These findings provide novel approaches for the exploration of cancer mechanism.

Human mitogen-activated protein kinase kinase 7(*MKK7*, also known as *MAP2K7*, MIM: 603014) belongs to the *MAP* kinase kinase family, and is identified as a tumor suppressor gene [11]. Evidence has demonstrated that *MKK7* serves as a critical signal transducer involved in several cancer-related signaling pathways and genes, and thus participates in regulating cells proliferation, differentiation and apoptosis [12–14]. *MKK7* deletion in mice caused distinct phenotypic abnormalities[15], whereas expression of *MKK7* could inhibit lung cancer cells development[16]. In addition, several studies also indicated that *MKK7* acts as a suppressor in tumors migration, invasion and metastasis [17–19].

Human *MKK7* gene is located at chromosome 19p13.3-p13.2, a region spanning over a fragile site associated with various human diseases [20, 21]. A study reported that the somatic mutations and loss of heterozygosity at 19p13.2 commonly existed in lung cancer [22]. Furthermore, another study showed that several non-synonymous somatic mutations of the *MKK7* gene also occurred and were associated with colorectal cancer predisposition [23]. Nevertheless, it is still molecularly unexplained how these rare variants implicated in cancer initiation and development. Therefore, in the current study, we test the hypothesis that the rare variants in *MKK7* might be associated with lung cancer risk and prognosis by disturbing the biological functions of MKK7.

Based on three independent case-control studies, we genotyped five rare SNPs in *MKK7* (i.e., rs28395770G>A: p.Glu116Lys, rs56316660A>G: p.Asn118Ser, rs56106612C>T: p.Arg138Cys, rs55800262G>A: p.Ala195Thr and rs1053566 C>T: p.Leu259Phe) and investigated their associations with lung cancer risk, metastasis and prognosis. The biological effects of those promising rare variants on lung cancer were further assessed by a series of functional experiments.

## Results

### Characteristics of the study populations

The demographic distributions of the three study populations are described in **Table 1.** Consistently, no significant deviations were observed in distributions of age, sex, drinking and family cancer history from the cases to controls in all the studied sets (*P* >0.05 for all), except for smoking status (*P* < 0.05). These variables were further adjusted in the multivariate logistic regression model to control possible confounding on the main effects of the studied polymorphisms. The histological types and clinical stages of the cases were also enumerated in **Table 1**. In addition, we recalculated the samples size based on population sources. There were 3005 cases and 3013 healthy controls in Guangzhou area, 2011 cases and 2168 cancer-free controls in Suzhou area.

**Table 1:** Frequency distributions of selected variables in lung cancer patients and cancer-free controls.

	Discovery set			Validation set I			Validation set II		
Variables	Case (n = 1559) n(%)	Control (n = 1679) n(%)	*P* ^a^	Case (n = 1949) n(%)	Control (n = 1957) n(%)	*P* ^a^	Case (n = 1508) n(%)	Control (n = 1545) n(%)	*P* ^a^
Age (years)									
≤ 60	809(51.9)	877(52.2)	0.846	1086(55.7)	1064(54.4)	0.396	796(52.8)	819(53.0)	0.901
> 60	750(48.1)	802(47.8)		863(44.3)	893(45.6)		712(47.2)	726(47.0)	
Sex									
Male	1091(70.0)	1185(70.6)	0.710	1391(71.4)	1398(71.4)	0.964	1083(71.8)	1086(70.3)	0.353
Female	468(30.0)	494(29.4)		558(28.6)	559(28.6)		425(28.2)	459(29.7)	
Family history of cancer									
Yes	129(8.3)	147(8.8)	0.625	155(8.0)	166(8.5)	0.547	119(7.9)	112(7.3)	0.502
No	1430(91.7)	1532(91.2)		1794(92.0)	1791(91.5)		1389(92.1)	1433(92.7)	
Family history of lung cancer									
Yes	52(3.3)	43(2.6)	0.192	46(2.4)	51(2.6)	0.638	39(2.6)	30(1.9)	0.231
No	1507(96.7)	1636(97.4)		1894(97.6)	1906(97.4)		1469(97.4)	1515(98.1)	
Smoking status									
Yes	824(52.8)	765(45.6)	3.37×10^−5^	1036(53.2)	952(48.7)	0.005	929(61.6)	655(42.4)	2.38×10^−26^
No	735(47.2)	914(54.4)		913(46.8)	1005(51.3)		579(38.4)	890(57.6)	
Pack-years smoked									
≥20	624(40.0)	479(28.5)	1.94×10^−11^	785(40.3)	496(25.4)	9.54×10^−29^	776(51.5)	463(30.0)	4.66×10^−33^
<20	200(12.8)	286(17.0)		251(12.9)	456(23.3)		153(10.1)	192(12.4)	
0	735(47.2)	914(54.5)		913(46.8)	1005(51.3)		579(38.4)	890(57.6)	
Drinking status									
Yes	293(18.8)	34220.4)	0.259	416(21.3)	426(21.8)	0.748	263(17.4)	286(18.5)	0.441
No	1266(81.2)	1337(79.7)		1533(78.7)	1531(78.2)		1245(82.6)	1259(81.5)	
Histological types									
Adenocarcinoma	615(39.4)			861(44.2)			818(54.2)		
Squamous cell carcinoma	527(33.8)			594(30.5)			444(29.5)		
Large cell carcinoma	66(4.2)			89(4.6)			35(2.3)		
Small cell lung cancer	193(12.4)			264(13.5)			161(10.7)		
Other carcinomas ^b^	158(10.2)			141(7.2)			50(3.3)		
Stages									
I	200(12.8)			207(10.6)			185(12.3)		
II	147(9.5)			225(11.6)			166(11.0)		
III	490(31.4)			593(30.4)			467(31.0)		
IV	722(46.3)			924(47.4)			690(45.7)		

^***a***^ *P* values for a χ^2^ test.

^***b***^ Mixed-cell or undifferentiated carcinoma.

### Associations between the *MKK7* rare SNPs and lung cancer risk

**Table 2** summarized the genotype distributions of the studied *MKK7* rare SNPs and their associations with lung cancer risk. In the discovery set, we found a significant frequency deviation between the cases and controls (exact *P* = 4.12×10^−12^) in p.Glu116Lys rare polymorphism. Compared to individuals with 116Glu/Glu genotype, the carriers with Lys/Glu heterozygote harbored a 3.33-fold increased risk of lung cancer (odd ratio [OR] = 3.33, 95% confidence interval [CI] = 2.29–4.86), and carriers with Lys/Lys variant genotype exerted a much higher cancer risk (OR = 3.94, 95% CI = 1.09–14.3). When combined with variant genotypes, they (Lys/Glu+Lys/Lys) also contributed a pernicious impact on lung cancer risk (OR = 3.38, 95% CI = 2.35–4.85), conforming to the fitted genetic model with the smallest akaike information criterion (AIC = 4415.3). However, we did not receive any association between other rare SNPs and lung cancer risk.

We further confirmed the above associations in another two validation sets, and obtained consistent results. The p.116Lys variants genotypes (Lys/Glu+Lys/Lys) exerted a 3.52-fold increased risk of lung cancer (OR = 3.52, 95% CI = 2.54–4.89) in validation set I, and a 2.87-fold increased risk of lung cancer (OR = 2.87, 95% CI = 2.04–4.04) in validation set II. Because the homogeneity test showed that the association in the above three sets was homogeneous (*P* = 0.711), we then merged the three populations to increase the study power, and found that the compared with Glu/Glu common genotype, the carrier with Lys/Glu or Lys/Lys had a remarkably adverse effects on lung cancer risk (OR = 3.23, 95% CI = 2.62–3.98; OR = 3.75, 95% CI = 2.09–6.71; respectively). Similarly, the Lys (Lys/Glu+Lys/Lys) variants also had a 3.29-fold increased risk of lung cancer under the dominant model (OR = 3.29, 95% CI = 2.70–4.01). The heritability test indicated that the p.Glu116Lys rare variant could explain about 2.16% of lung cancer heritability.

**Table 2:** Associations between *MKK7* rare polymorphisms and lung cancer risk.

*MKK7* SNPs	Discovery set			Validation set I			Validation set II			Pooled analysis ^b^		
	Case n (%)	Control n (%)	Adjusted OR (95% CI) ^*a*^	Case n (%)	Control n (%)	Adjusted OR (95% CI) ^a^	Case n (%)	Control n (%)	Adjusted OR (95% CI) ^a^	Case n (%)	Control n (%)	Adjusted OR (95% CI) ^a^
p. Glu116Lys (rs28395770G>A)	1559	1679		1949	1957		1508	1545		5016	5181	
Codominant model ^*c*^			4417.3			5315.2			4037.6			13824.3
Glu/Glu	1437(92.2)	1638(97.6)	1.00 (ref.)	1786(91.6)	1908(97.5)	1.00 (ref.)	1373(91.0)	1495(96.8)	1.00 (ref.)	4596(91.6)	5041(97.3)	1.00 (ref.)
Lys/Glu	111(7.1)	38(2.3)	**3.33(2.29–4.86)**	140(7.2)	42(2.1)	**3.53(2.48–5.01)**	119(7.9)	45(2.9)	**2.83(1.97–4.05)**	370(7.4)	125(2.4)	**3.23(2.62–3.98)**
Lys/Lys	11(0.7)	3(0.1)	**3.94(1.09–14.3)**	23(1.2)	7(0.4)	**3.52(1.50–8.26)**	16(1.1)	5(0.3)	**3.26(1.16–9.14)**	50(1.0)	15(0.3)	**3.75(2.09–6.71)**
Fisher test *P* value			**4.12×10**^**−12**^			**7.99×10**^**−16**^			**8.98×10**^**−11**^			**1.09×10**^**−36**^
Additive model ^c^			4416.9			5317.9			4037.5			13829.6
Lys/Lys *vs*. Lys/Glu *vs*. Glu/Glu			**3.00(2.14–4.21)**			**2.85(2.14–3.81)**			**2.49(1.83–3.38)**			**2.80(2.34–3.34)**
Dominant model ^*c*^			4415.3			5313.2			4035.7			13822.5
Glu/Glu	1437(92.2)	1638(97.6)	1.00 (ref.)	1786(91.6)	1908(97.5)	1.00 (ref.)	1373(91.0)	1495(96.8)	1.00 (ref.)	4596(91.6)	5041(97.3)	1.00 (ref.)
Lys/Lys+Lys/Glu	122(7.8)	41(2.4)	**3.38(2.35–4.85)**	163(8.4)	49(2.5)	**3.52(2.54–4.89)**	135(9.0)	50(3.2)	**2.87(2.04–4.04)**	420(8.4)	140(2.7)	**3.29(2.70–4.01)**
p.Asn118Ser (rs56316660A>G)												
Codominant model ^*c*^			4464.1			5381.6			4077.2			13982.3
Asn/Asn	1535(98.5)	1664(99.1)	1.00 (ref.)	1921(98.6)	1933(98.8)	1.00 (ref.)	1490(98.8)	1532(99.2)	1.00 (ref.)	4946(98.6)	5129(99.0)	1.00 (ref.)
Ser/Asn	19(1.2)	12(0.7)	1.65(0.79–3.41)	22(1.1)	19(1.0)	1.23(0.66–2.30)	15(1.0)	11(0.7)	1.56(0.70–3.48)	56(1.2)	42(0.8)	1.39(0.93–2.08)
Ser/Ser	5(0.3)	3(0.2)	2.24(0.53–9.47)	6(0.3)	5(0.2)	1.13(0.34–3.75)	3(0.2)	2(0.1)	1.44(0.23–9.14)	14(0.3)	10(0.2)	1.59(0.70–3.60)
Fisher test *P* value			0.224			0.813			0.612			0.190
Additive model ^c^			4462.1			5379.7			4075.4			13980.4
Ser/Ser *vs*. Ser/Asn *vs*. Asn/Asn			1.57(0.94–2.63)			1.14(0.74–1.77)			1.40(0.76–2.58)			1.32(0.98–1.77)
Dominant model ^*c*^			4462.2			5379.6			4075.2			13980.4
Asn/Asn	1535(98.5)	1664(99.1)	1.00 (ref.)	1921(98.6)	1933(98.8)	1.00 (ref.)	1490(98.8)	1532(99.2)	1.00 (ref.)	4946(98.6)	5129(99.0)	1.00 (ref.)
Ser/Ser+Ser/Asn	24(1.5)	15(0.9)	1.76(0.91–3.37)	28(1.4)	24(1.2)	1.21(0.70–2.11)	18(1.2)	13(0.8)	1.54(0.74–3.22)	70(1.5)	52(1.0)	1.43(0.98–2.05)
p.Arg138Cys (rs56106612C>T)												
Codominant model ^*c*^			4464.3			5381.3			4077.7			13982.6
Arg/Arg	1539(98.7)	1668(99.3)	1.00 (ref.)	1927(98.9)	1940(99.1)	1.00 (ref.)	1491(98.9)	1532(99.1)	1.00 (ref.)	4957(98.8)	5140(99.2)	1.00 (ref.)
Cys/Arg	15(1.0)	9(0.5)	1.70(0.74–3.91)	18(0.9)	14(0.7)	1.27(0.63–2.56)	15(1.0)	12(0.8)	1.18(0.54–2.56)	48(1.0)	35(0.7)	1.35(0.87–2.10)
Cys/Cys	5(0.3)	2(0.1)	2.42(0.47–12.6)	4(0.2)	3(0.2)	1.54(0.34–6.93)	2(0.1)	1(0.1)	2.75(0.23–32.8)	11(0.2)	6(0.1)	1.89(0.70–5.16)
Fisher test *P* value			0.192			0.688			0.627			0.131
Additive model ^c^			4462.4			5379.3			4075.9			13980.7
Cys/Cys *vs*. Cys/Arg *vs*. Arg/Arg			1.63(0.90–2.94)			1.25(0.75–2.10)			1.30(0.68–2.50)			1.36(0.98–1.90)
Dominant model ^*c*^			4462.5			5379.4			4076.2			13981.0
Arg/Arg	1539(98.7)	1668(99.3)	1.00 (ref.)	1927(98.9)	1940(99.1)	1.00 (ref.)	1491(98.9)	1532(99.1)	1.00 (ref.)	4957(98.8)	5140(99.2)	1.00 (ref.)
Cys/Cys+Cys/Arg	20(1.3)	11(0.6)	1.83(0.87–3.85)	22(1.1)	17(0.9)	1.31(0.69–2.49)	17(1.1)	13(0.9)	1.28(0.61–2.68)	59(1.2)	41(0.8)	1.43(0.96–2.14)
p.Ala195Thr (rs55800262G>A)												
Codominant model ^*c*^			4464.1			5381.7			4077.2			13982.6
Ala/Ala	1537(98.6)	1666(99.2)	1.00 (ref.)	1930(99.0)	1941(99.2)	1.00 (ref.)	1487(98.6)	1529(98.9)	1.00 (ref.)	4954(98.8)	5136(99.1)	1.00 (ref.)
Thr/Ala	18(1.2)	11(0.7)	1.75(0.82–3.73)	13(0.7)	12(0.6)	1.16(0.52–2.56)	16(1.1)	12(0.8)	1.51(0.70–3.27)	47(0.9)	35(0.7)	1.43(0.92–2.24)
Thr/Thr	4(0.3)	2(0.1)	2.18(0.40–12.0)	6(0.3)	4(0.2)	1.39(0.39–4.97)	5(0.3)	4(0.3)	1.47(0.37–5.90)	15(0.3)	10(0.2)	1.49(0.67–3.35)
Fisher test *P* value			0.198			0.762			0.703			0.189
Additive model ^c^			4462.2			5379.7			4075.4			13980.9
Thr/Thr vs. Thr/Ala vs. Ala/Ala			1.63(0.92–2.89)			1.17(0.71–1.93)			1.34(0.80–2.25)			1.32(0.97–1.78)
Dominant model ^*c*^			4462.2			5379.7			4075.2			13980.6
Ala/Ala	1537(98.6)	1666(99.2)	1.00 (ref.)	1930(99.0)	1941(99.2)	1.00 (ref.)	1487(98.6)	1529(98.9)	1.00 (ref.)	4954(98.8)	5136(99.1)	1.00 (ref.)
Thr/Thr+Thr/Ala	22(1.5)	13(0.8)	1.82(0.91–3.63)	19(1.0)	16(0.8)	1.22(0.62–2.39)	21(1.4)	16(1.1)	1.50(0.76–2.95)	62(1.2)	45(0.9)	1.45(0.98–2.14)
p.Leu259Phe (rs1053566 C>T)												
Codominant model ^*c*^			4464.2			5381.7			4077.4			13982.4
Leu/Leu	1543(99.0)	1670(99.4)	1.00 (ref.)	1936(99.3)	1946(99.4)	1.00 (ref.)	1491(98.9)	1532(99.2)	1.00 (ref.)	4970(99.1)	5148(99.3)	1.00 (ref.)
Phe/Leu	10(0.6)	6(0.4)	1.89(0.68–5.25)	9(0.5)	8(0.4)	1.13(0.43–2.94)	14(0.9)	11(0.7)	1.44(0.63–3.28)	33(0.7)	25(0.5)	1.45(0.85–2.44)
Phe/Phe	6 (0.4)	3(0.2)	2.28(0.57–9.18)	4(0.2)	3(0.2)	1.52(0.33–6.92)	3(0.2)	2(0.1)	1.82(0.28–11.7)	13(0.3)	8(0.2)	1.83(0.75–4.47)
Fisher test *P* value			0.300			0.847			0.679			0.250
Additive model ^c^			4462.3			5379.7			4075.4			13980.4
Phe/Phe *vs*. Phe/Leu *vs*. Leu/Leu			1.63(0.91–2.91)			1.19(0.66–2.16)						1.39(0.99–1.96)
Dominant model ^*c*^			4462.2			5379.8			4075.5			13980.6
Leu/Leu	1543(99.0)	1670(99.4)	1.00 (ref.)	1936(99.3)	1946(99.4)	1.00 (ref.)	1491(98.9)	1532(99.2)	1.00 (ref.)	4970(99.1)	5148(99.3)	1.00 (ref.)
Phe/Phe+Phe/Leu	16(1.0)	9(0.6)	2.02(0.89–4.61)	13(0.7)	11(0.6)	1.23(0.54–2.76)	17(1.1)	13(0.8)	1.50(0.70–3.18)	44(1.0)	33(0.7)	1.54(0.98–2.42)

^***a***^ Data were calculated by exact logistic regression adjusted for selected surrounding factors.

^*b*^ The data comprised the discovery set and two validation sets.

^*c*^ Akaike information criterion (AIC) value.

### Stratified analysis of association between *MKK7* p.Glu116Lys and lung cancer risk

In stratification analysis, as is presented in **Table 3**, no deviation of p.116Lys variants on cancer risk was observed in most subgroups except for the strata of clinical stages (*P* = 0.016). We further evaluated the relationships between *MKK7* p.Glu116Lys and lung cancer progression, and found that p.Glu116Lys was significantly associated with pejorative clinical stages (*P* <0.001, shown in **S1 Table**). As is revealed in **S1 Table**, the patients with 116Lys variants had increased probability of progressing to IV stage (OR = 1.69, 95% CI = 1.28–2.24). Likewise, the frequency of p.116Lys adverse genotypes elevated continuously along with the risk of lymphatic metastasis extent at diagnosis (5.8% for 0, 8.5% for 1, 8.7% for 2, and 10. 8% for 3), and with the distal metastasis extent at diagnosis (7.0% for 0, 9.9% for 1). In brief, patients with 116Lys variants were more likely to have metastasis (either nodal or distal metastasis) than those with Glu/Glu genotype (OR = 1.84, 95% CI = 1.34–2.53).

**Table 3:** Stratified analysis of association between p.Glu116Lys and lung cancer risk.

	Cases (*n* = 5016)		Controls (*n* = 5181)		Adjusted OR (95% CI) ^a^		
	Glu/Glu*n*.(%)	Lys/Glu+Lys/Lys*n*.(%)	Glu/Glu*n*.(%)	Lys/Glu+Lys/Lys*n*.(%)	Lys/Glu+Lys/Lys *vs*. Glu/Glu	*P*_homo_ ^b^	*P*_inter_ ^c^
Age (years)						0.167	0.562
≤ 60	2474(91.9)	217(8.1)	2679(97.1)	81(2.9)	**2.90(2.23–3.78)**		
> 60	2122(91.3)	203(8.7)	2362(97.6)	59(2.4)	**3.82(2.83–5.15)**		
Sex						0.101	0.739
Male	3270(91.7)	295(8.3)	3561(97.1)	1008(2.9)	**2.99(2.38–3.75)**		
Female	1326(91.4)	125(8.6)	1480(97.9)	32(2.1)	**4.18(2.81–6.23)**		
Smoking status						0.511	0.773
Ever	2557(91.7)	232(8.3)	2304(97.1)	68(2.9)	**3.03(2.30–4.00)**		
Never	2039(91.6)	188(8.4)	2737(97.4)	72(2.6)	**3.48(2.63–4.60)**		
Drinking status						0.795	0.878
Ever	891(91.7)	81(8.3)	1024(97.2)	30(2.8)	**3.08(1.99–4.77)**		
Never	3705(91.6)	339(8.4)	4017(97.3)	110(2.7)	**3.33(2.67–4.16)**		
Family history of cancer						0.756	0.769
Yes	371(92.1)	32(7.9)	413(97.2)	12(2.8)	**2.88(1.45–5.74)**		
No	4225(91.6)	388(8.4)	4628(97.3)	128(2.7)	**3.32(2.70–4.06)**		
Family history of lung cancer						0.698	0.281
Yes	124(90.5)	13(9.5)	121(97.6)	3(2.4)	**4.71(1.20–18.5)**		
No	4463(91.6)	407(8.4)	4920(97.3)	137(2.7)	**3.27(2.67–3.99)**		
Population sources						0.350	0.163
Guangzhou	2762(91.9)	243(8.1)	2941(97.6)	72(2.4)	**3.57(2.73–4.67)**		
Suzhou	1834(91.2)	177(8.8)	2100(96.9)	68(3.1)	**2.93(2.19–3.92)**		
Stages						**0.016**	
I+II	1061(93.9)	69(6.1)			**2.30(1.71–3.10)**		
III	1430(92.3)	120(7.7)	5041(97.3)	140(2.7)	**3.11(2.41–4.00)**		
IV	2105(90.1)	231(9.9)			**3.91(3.14–4.86)**		
Histological types						0.478	
Adenocarcinoma	2088(91.0)	206(9.0)			**3.59(2.87–4.49)**		
Squamous cell carcinoma	1429(91.3)	136(8.7)			**3.39(2.64–4.36)**		
Large cell carcinoma	180(94.7)	10(5.3)	5041(97.3)	140(2.7)	**1.98(1.03–3.83)**		
Small cell lung cancer	575(93.0)	43(7.0)			**2.63(1.84–3.75)**		
Other carcinomas ^d^	324(92.8)	25(7.2)			**2.74(1.76–4.26)**		

^*a*^ ORs were adjusted for age, sex, and smoking status, and alcohol use, family history of cancer in a logistic regression model.

^*b*^ *P* value of homogeneity test between strata for the related ORs of *MKK7* Glu116Lys (Lys/Glu+Lys/Lys *vs*. Glu/Glu genotypes).

^*c*^ *P* value of test for the multiplicative interaction between *MKK7* Glu116Lys genotypes and selected variables on cancer risk in logistic regression models.

^*d*^ Mixed-cell or undifferentiated carcinoma.

### Associations between the combinations of *MKK7* rare SNPs and lung cancer risk

We further evaluated the associations between the combined types of those selected rare SNPs and lung cancer risk. As is shown in **S2 Table**, the individuals with only p.Glu116Lys variant was associated with lung cancer susceptibility (exact *P* = 1.18×10^−33^), accompanying by a 3.24-fold increased cancer risk (OR = 3.24, 95% CI = 2.64–3.97), which was best fitted for the heredity model (AIC value = 13840.2). It achieved 100% study power and yielded a value of 0.000 with a 0.001 prior probability lower than the preset FPRP-level criterion 0.20, suggesting that this finding is noteworthy. Individuals with a combination of p.Glu116Lys and p.Asn118Ser variant genotypes also had an increased risk of lung cancer (OR = 3.16, 95% CI = 1.02–9.76), but it achieved only 66.7% moderate power and a 0.985 FPRP value at a 0.001 prior probability, which is higher than the preset criterion 0.20. Furthermore, we also used the SKAT method to test combined genotypes associated with lung cancer risk, and found that only those combinations containing the p.Glu116Lys rare variation had prominent relevancies with lung cancer risk(*P* <0.01 for all). All these results indicated that among all of the *MKK7* five rare polymorphisms, the p.Glu116Lys contributed the main effect on lung cancer risk. A serial of experiments was further conducted to decipher the biological mechanisms of p.Glu116Lys on lung cancer.

### Associations of the *MKK7* rare SNPs with lung cancer prognosis

The distributions of demographic and clinical characteristics in the three datasets are presented in **S3 Table**. The Log-rank test and univariate Cox analysis revealed that patients with characteristics including ≥60, smoking or advanced stage had a significantly shorter median survival time (MST) and an increased death risk (*P* <0.05 for all). In contrast, the female patients, and those patients suffering from surgical operations, chemotherapy or radiotherapy prolonged survival time and had a more benignant prognosis (shown in **S3 Table**).

**Fig 1:**
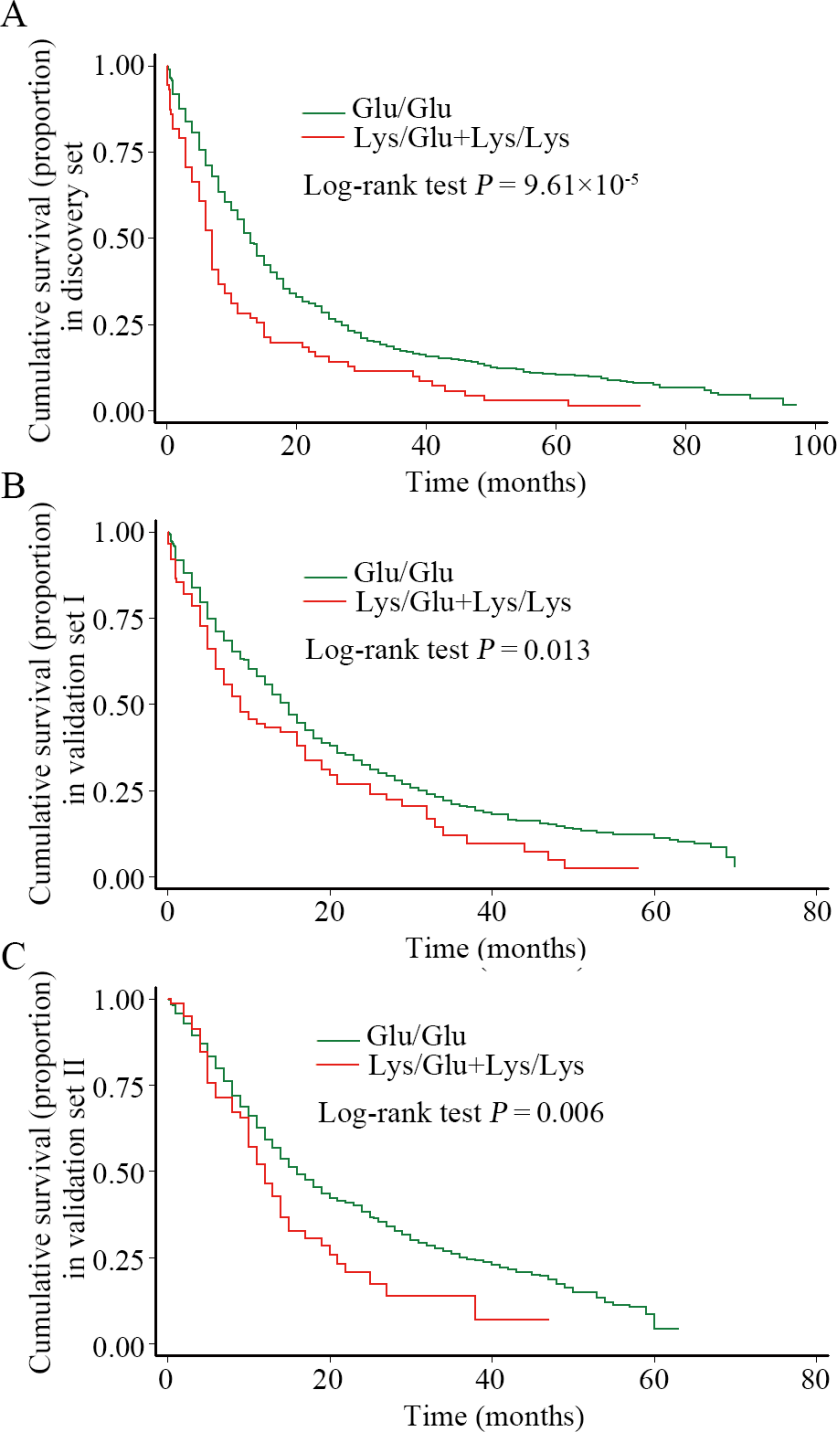
Kaplan-Meier (KM) survival curves for lung cancer patients carrying the different *MKK7* p.Glu116Lys genotypes. The *P* value was calculated by the Log-rank test. (A). KM curves of patients from the discovery set; (B). KM curves of patients from validation set I; (C). KM curves of patients from validation set II.

The relevancies between the *MKK7* rare SNPs and lung cancer outcomes are shown in **Table 4**. In the discovery set, compared with Glu/Glu genotype, the patients with p.116Glu/Lys heterozygote had a significantly shorter MST (7 months *vs*. 13 months; Log-rank test *P* = 6.19×10^−5^) and a higher death risk (hazard ratio [HR] = 1.69, 95% CI = 1.31–2.19). Multivariate proportional hazards regression analysis indicated that this rare variant appeared an undesirable survival of lung cancer under the additive genetic model (HR = 1.63, 95% CI = 1.31–2.03). Congruously, the 116Lys (Lys/Glu+Lys/Lys) variants exerted a poor prognosis (HR = 1.73, 95% CI = 1.35–2.21) and a shorter MST (7 months *vs*. 13 months; Log-rank test *P* = 9.61×10^−5^; **Fig 1A)**, while compared to the Glu/Glu wild-genotype. However, for other rare SNPs, no significant associations with lung cancer survival were found.

**Table 4:** Associations between *MKK7* rare polymorphisms and lung cancer survival in the three populations.

		Discovery set					Validation set I					Validation set II					Merged set			
Gene SNPs	Patients.n(%)	Death	MST (months)	Log-rank *P* value	HR (95%CI) ^a^	Patients.n(%)	Death	MST (months)	Log-rank *P* value	HR (95%CI) ^a^	Patients.n(%)	Death	MST (months)	Log-rank *P* value	HR (95%CI) ^a^	Patients.n(%)	Death	MST (months)	Log-rank *P* value	HR (95%CI) ^a^
*MKK7*	908	841				1027	766				971	672				2906	2279			
p.Glu116Lys																				
Glu/Glu	837(92.2)	771	13	**6.19×10**^**−5**^	1.00 (ref.)	939(91.4)	692	15	**0.037**	1.00 (ref.)	892(91.9)	620	16	**0.010**	1.00 (ref.)	2668(91.8)	2083	14	**2.61×10**^**−7**^	1.00 (ref.)
Lys/Glu	65(7.2)	64	7		**1.69(1.31–2.19)**	75(7.3)	63	9		**1.43(1.10–1.85)**	70(7.2)	44	12		**1.41(1.04–1.92)**	210(7.2)	171	10		**1.49(1.27–1.74)**
Lys/Lys	6(0.6)	6	6		2.24(0.99–5.06)	13(1.3)	11	7		**1.99(1.09–3.64)**	9(0.9)	8	9		**2.34(1.15–4.75)**	28(1.0)	25	7		**1.94(1.31–2.89)**
Additive model ^*b*^																				
Lys/Lys vs. Lys/Glu vs. Glu/Glu					**1.63(1.31–2.03)**					**1.42(1.16–1.74)**					**1.46(1.15–1.85)**					**1.46(1.29–1.66)**
Dominant model ^*b*^																				
Glu/Glu	837(92.2)	771	13	**9.61×10**^**−5**^	1.00 (ref.)	939(91.4)	692	15	**0.013**	1.00 (ref.)	892(91.9)	620	16	**0.006**	1.00 (ref.)	2669(91.8)	2084	14	**1.03×10**^**−6**^	1.00 (ref.)
Lys/Lys+Lys/Glu	71(7.8)	70	7		**1.73(1.35–2.21)**	88(8.6)	74	9		**1.49(1.17–1.90)**	79(8.1)	52	12		**1.50(1.13–2.00)**	237(8.2)	195	9		**1.53(1.32–1.78)**
p.Asn118Ser																				
Asn/Asn	894(98.5)	828	12	0.856	1.00 (ref.)	1010(98.3)	752	14	0.863	1.00 (ref.)	956(98.5)	660	16	0.084	1.00 (ref.)	2860(98.4)	2240	14	0.501	1.00 (ref.)
Ser/Asn	12(1.3)	11	10		1.35(0.74–2.46)	15(1.5)	12	16		1.00(0.56–1.77)	13(1.3)	11	9		1.59(0.87–2.91)	40(1.4)	34	12		1.26(0.90–1.77)
Ser/Ser	2(0.2)	2	13		1.77(0.44–7.11)	2(0.2)	12	14		1.66(0.41–6.65)	2(0.2)	1	28		0.56(0.08–4.04)	6(0.2)	5	13		1.34(0.56–3.22)
Additive model ^*b*^																				
Ser/Ser vs. Ser/Asn vs. Asn/Asn					1.34(0.85–2.12)					1.09(0.69–1.73)					1.17(0.73–1.88)					1.22(0.93–1.59)
Dominant model ^*b*^																				
Asn/Asn	894(98.5)	828	12	0.620	1.00 (ref.)	1010(98.3)	752	14	0.850	1.00 (ref.)	956(98.5)	660	16	0.183	1.00 (ref.)	2860(98.4)	2240	14	0.267	1.00 (ref.)
Ser/Ser+Ser/Asn	14(1.5)	13	11		1.40(0.80–2.44)	17(1.7)	14	16		1.06(0.62–1.80)	15(1.5)	12	9		1.38(0.78–2.46)	46(1.6)	39	12		1.27(0.92–1.74)
p.Arg138Cys																				
Arg/Arg	901(99.2)	834	12	0.586	1.00 (ref.)	1015(98.8)	757	14	0.931	1.00 (ref.)	961(99.0)	664	16	0.142	1.00 (ref.)	2877(99.0)	2255	14	0.444	1.00 (ref.)
Cys/Arg	6(0.7)	6	12		1.13(0.50–2.53)	9(0.9)	7	7		1.39(0.66–2.96)	9(0.9)	7	11		1.16(0.55–2.46)	24(0.8)	20	11		1.20(0.77–1.87)
Cys/Cys	1(0.1)	1	8		1.49(0.21–10.6)	3(0.3)	2	12		1.05(0.26–4.22)	1(0.1)	1	5		5.99(0.84–43.1)	5(0.2)	4	8		1.48(0.55–3.96)
Additive model ^*b*^																				
Cys/Cys vs. Cys/Arg vs. Arg/Arg					1.16(0.72–2.19)					1.16(0.71–1.90)					1.39(0.74–2.64)					1.21(0.87–1.68)
Dominant model ^*b*^																				
Arg/Arg	901(99.2)	834	12	0.374	1.00 (ref.)	1015(98.8)	757	14	0.725	1.00 (ref.)	981(99.0)	664	16	0.392	1.00 (ref.)	2877(99.0)	2255	14	0.242	1.00 (ref.)
Cys/Cys+Cys/Arg	7(0.8)	7	11		1.17(0.55–2.47)	12(1.2)	9	10		1.30(0.67–2.52)	10(1.0)	8	9.5		1.29(0.64–2.61)	29(1.0)	24	11		1.24(0.83–1.86)
p.Ala195Thr																				
Ala/Ala	898(98.9)	831	13	0.313	1.00 (ref.)	1017(99.0)	758	14	0.823	1.00 (ref.)	956(98.5)	662	15	0.095	1.00 (ref.)	2871(98.8)	2251	14	0.407	1.00 (ref.)
Thr/Ala	9(1.0)	9	10		1.53(0.79–2.96)	7(0.7)	6	11		1.41(0.63–3.18)	12(1.2)	7	36		0.49(0.23–1.04)	28(1.0)	22	11		0.95(0.63–1.45)
Thr/Thr	1(0.1)	1	8		1.78(0.25–12.7)	3(0.3)	2	5		1.21(0.30–4.87)	3(0.3)	3	13		1.57(0.50–4.97)	7(0.2)	6	8		1.62(0.73–3.61)
Additive model ^*b*^																				
Thr/Thr vs. Thr/Ala vs. Ala/Ala					1.46(0.85–2.51)					1.21(0.72–2.03)					0.76(0.46–1.27)					1.09(0.81–1.48)
Dominant model ^*b*^																				
Ala/Ala	898(98.9)	831	13	0.141	1.00 (ref.)	1017(99.0)	758	14	0.580	1.00 (ref.)	958(98.5)	660	15	0.125	1.00 (ref.)	2871(98.8)	2251	14	0.793	1.00 (ref.)
Thr/Thr+Thr/Ala	10(1.1)	10	9.5		1.55(0.82–2.90)	10(1.0)	8	9		1.35(0.67–2.74)	15(1.5)	10	24		0.62(0.33–1.17)	35(1.2)	28	11		1.05(0.72–1.52)
p.Leu259Phe																				
Leu/Leu	900(99.1)	833	12	0.338	1.00 (ref.)	1017(99.0)	759	14	0.457	1.00 (ref.)	964(99.3)	665	16	0.194	1.00 (ref.)	2881(99.1)	2257	14	0.090	1.00 (ref.)
Phe/Leu	6(0.7)	6	14.5		1.15(0.51–2.58)	7(0.7)	4	29		0.73(0.27–1.96)	6(0.6)	6	11.5		1.61(0.72–3.62)	19(0.7)	16	15		1.15(0.70–1.88)
Phe/Phe	2(0.2)	2	6.5		2.39(0.59–3.60)	3(0.3)	3	8		1.96(0.62–6.16)	1(0.1)	1	9		3.09(0.43–22.3)	6(0.2)	6	8		2.25(0.98–5.08)
Additive model ^*b*^																				
Phe/Phe vs. Phe/Leu vs. Leu/Leu					1.34(0.77–2.32)					1.12(0.66–1.91)					1.67(0.88–3.14)					1.33(0.97–1.84)
Dominant model ^*b*^																				
Leu/Leu	900(99.1)	833	12	0.429	1.00 (ref.)	1017(99.0)	759	14	0.892	1.00 (ref.)	964(99.3)	665	16	0.083	1.00 (ref.)	2881(99.1)	2257	14	0.153	1.00 (ref.)
Phe/Phe+ Phe/Leu	8(0.9)	8	12		1.32(0.66–2.67)	10(1.0)	7	15.5		1.00(0.47–2.11)	7(0.7)	7	10		1.73(0.82–3.67)	25(0.9)	22	13		1.33(0.87–2.02)

Abbreviations: MST, median survival time; HR, hazard ratio; ref., reference. Bold type: statistically significant, *P* < 0.05.

^*a*^ The Cox regression analysis was adjusted for age, sex, smoking, stage, histology, surgery, chemo-therapy, and radio therapy status

The associations between *MKK7* rare SNPs and prognosis of lung cancer were further verified in other two validation sets. In those two datasets, when compared with the Glu/Glu genotype, patients with Lys/Glu genotype had a decreased MST (validation set I: 9 months *vs*. 15 months, Log-rank test *P* = 0.033; validation set II: 12 months *vs*. 16 months, Log-rank test *P* = 0.031) and had shown an increased death risk (validation set I: HR = 1.43, 95% CI = 1.10–1.85; validation set II: HR = 1.41, 95% CI = 1.04–1.92); those patients carrying Lys/Lys homozygote also exerted a pernicious cancer prognosis (validation set I: HR = 1.99, 95% CI = 1.09–3.64; validation set II: HR = 2.34, 95% CI = 1.15–4.75), along with a shorter MST (validation set I: 7 months *vs*. 15 months, Log-rank test *P* = 0.048; validation set II: 9 months *vs*. 16 months, Log-rank test *P* = 0.026). Similarly, the patients with p.116Lys (Lys/Lys+Lys/Glu) variants presented a shorter MST (validation set I: 9 months vs. 15 months, Log-rank test *P* = 0.013, **Fig 1B**; validation set II: 12 months vs. 16 months, Log-rank test *P* = 0.006, **Fig 1C**) and worse survival outcomes (validation set I: HR = 1.49, 95% CI = 1.17–1.90; validation set II: HR = 1.50, 95% CI = 1.13–2.00).

Pooled analysis of the three cohorts indicated that patients with Lys/Glu or Lys/Lys variant genotype harbored reduced 4 and 7 MST months (*P* = 2.61×10^−7^), coupling with a 149% (HR = 1.49, 95% CI = 1.27–1.74) and a 194% (HR = 1.94, 95% CI = 1.31–2.89) cancer death risk, respectively, while compared to patients with Glu/Glu genotype. Also, the p.116Lys (Lys/Lys+Lys/Glu) detrimental genotypes conferred a 5-months decreased in MST compared with that of Glu/Glu genotype (9 months *vs*. 14 months, Log-rank test *P* = 1.03×10^−6^) and had a 53% higher death risk (HR = 1.53, 95% CI = 1.32–1.78).

### Stratified analysis of the *MKK7* p.Glu116Lys and lung cancer survival

As is revealed in **Table 5**, although the strength of relevance represented by the HR values between the p.116Lys variants and lung cancer prognosis were different across a plurality of stratums, the homogeneity test showed that the difference was only significant in subgroups of clinical stage and distant metastasis (*P* values equal to 0.019 and 0.010, respectively). The unfavorable influence of the p.116Lys variants on cancer prognosis was more conspicuous in advanced stages (HR = 1.88, 95% CI = 1.53–2.30). The patients in the distant metastasis stage had 62% higher death risk than those without metastasis (HR: 1.88 *vs*. 1.26). We also found a remarkable modification effect between the clinical stage and the p.116Lys variants on lung cancer prognosis (*P* = 0.039).

**Table 5:** Stratification analysis of association between the *MKK7* p.Glu116Lys genotypes and lung cancer prognosis by selected variables.

Variables	Glu/Glu genotype		Lys/Lys+Lys/Glu genotypes		Adjusted HR (95% CI) ^a^	*P*_homo_ ^b^	*P*_inter_ ^c^
	Patients. n	Death. n(%)	Patients. n	Death. n(%)			
Age (years)						0.641	0.791
≤ 60	1345	1018(75.7)	125	97(77.6)	**1.51(1.23–1.87)**		
> 60	1323	1065(80.5)	113	99(87.6)	**1.62(1.31–1.99)**		
Sex						0.090	0.109
Male	1916	1501(78.3)	154	129(83.8)	**1.68(1.40–2.02)**		
Female	752	582(77.4)	84	67(79.8)	1.28(0.99–1.65)		
Family history of cancer						0.595	0.229
No	2453	1925(78.5)	220	184(83.6)	**1.56(1.34–1.82)**		
Yes	215	158(73.5)	18	12(66.7)	1.32(0.73–2.41)		
Family history of lung cancer						0.448	0.255
No	2587	2026(78.3)	231	192(83.1)	**1.55(1.34–1.80)**		
Yes	81	57(70.4)	7	4(57.1)	1.03(0.36–2.91)		
Smoking status						0.261	0.178
Never	1169	907(77.6)	113	92(81.4)	**1.40(1.13–1.74)**		
Ever	1499	1176(78.5)	125	104(83.2)	**1.66(1.35–2.03)**		
Drinking status						0.188	0.148
Never	2168	1691(78.0)	197	161(81.7)	**1.48(1.26–1.74)**		
Ever	500	392(78.4)	41	35(85.4)	**1.92(1.35–2.73)**		
Surgery						0.584	0.522
No	1665	1319(79.2)	132	108(81.8)	**1.49(1.22–1.82)**		
Yes	1003	764(76.2)	106	88(83.0)	**1.62(1.30–2.03)**		
Chemotherapy						0.837	0.848
No	938	778(82.9)	84	75(89.3)	**1.53(1.20–1.95)**		
Yes	1730	1305(75.4)	154	121(78.6)	**1.58(1.31–1.90)**		
Radiotherapy						0.157	0.357
No	1375	1041(75.7)	124	95(76.6)	**1.38(1.12–1.71)**		
Yes	1293	1042(80.6)	114	101(88.6)	**1.71(1.39–2.11)**		
Histological types						0.290	0.450
Adenocarcinoma	1230	923(75.0)	121	92(76.0)	**1.30(1.05–1.62)**		
Squamous cell carcinoma	812	652(80.3)	77	68(88.3)	**1.71(1.33–2.20)**		
Large cell carcinoma	104	84(80.8)	4	4(100.0)	2.67(0.90–7.90)		
Small cell lung cancer	321	268(83.5)	20	18(90.0)	**1.92(1.18–3.12)**		
Other ^d^	201	156(77.6)	16	14(87.5)	1.75(0.99–3.10)		
Population sources						0.769	0.803
Guangzhou	1478	1190(80.5)	133	118(88.7)	**1.52(1.26–1.84)**		
Suzhou	1190	893(75.0)	105	78(74.3)	**1.59(1.26–2.01)**		
Stages						**0.019**	**0.039**
I+II	564	392(69.5)	42	31(73.8)	1.12(0.77–1.62)		
III	871	697(80.0)	70	59(84.3)	1.31(1.00–1.71)		
IV	1233	994(80.6)	126	106(84.1)	**1.88(1.53–2.30)**		
Nodal metastasis						0.966	0.282
0	640	436(68.1)	44	29(65.9)	**1.52(1.04–2.23)**		
1	559	436(78.0)	58	47(81.0)	**1.38(1.02–1.88)**		
2	893	738(82.6)	69	59(85.5)	**1.50(1.15–1.97)**		
3	576	473(82.1)	67	61(91.0)	**1.52(1.15–1.99)**		
Distal metastasis						**0.010**	0.088
0	1435	1089(75.9)	112	90(80.4)	**1.26(1.01–1.57)**		
1	1233	994(80.6)	126	106(84.1)	**1.88(1.53–2.30)**		
Metastasis						0.555	0.282
No	440	291(66.1)	26	18(69.2)	1.30(0.79–2.13)		
Yes	2228	1792(80.4)	212	178(84.0)	**1.52(1.30–1.77)**		

Bold type: statistically significant, *P* < 0.05.

^*a*^ HRs were adjusted for age, smoking, stage, histology, surgery, chemo-therapy, and radio therapy status in a Cox regression model.

^*b*^ *P* value of homogeneity test between strata for the related ORs of p.Glu116Lys (Lys/Lys+Lys/Glu vs. Glu/Glu genotype).

^*c*^ *P* value of test for the multiplicative interaction between p.Glu116Lys genotypes and selected variables on cancer death in Cox regression models.

^*d*^ Mixed-cell or undifferentiated carcinoma.

### Associations between the combinations of *MKK7* rare SNPs and lung cancer prognosis

We further analyzed the associations between the combinational genotypes of *MKK7* rare SNPs and lung cancer prognosis. As is presented in **S4 Table**, combined-type of only p. Glu116Lys was significantly associated with cancer outcome. Patients only with Lys variants genotypes showed a shorter MST (9 months *vs*. 14 months, Log-rank test *P* = 4.01×10^−8^) and a higher death risk (HR = 1.58, 95% CI = 1.35–1.85) when compared with patients without those genotypes. This noticeable result achieved 100% study power and yielded a value of 0.000 at a 0.001 prior probability, which is lower than the preset FPRP-level criterion 0.20. Although the combination of the p.Glu116Lys and p.Asn118Ser was significantly associated with survival time using Log-rank test (*P* = 0.025), but it obliterated the relevance in the Cox regression analysis (HR = 1.74, 95% CI = 0.99–3.08) and obtained a FPRP value of 0.991 at a 0.001 prior probability higher than the preset criterion 0.20, suggesting that this result was likely to be untrustworthy.

### Effects of *MKK7* p.Glu116Lys on cellular proliferation, apoptosis, migration and invasion

**Fig 2:**
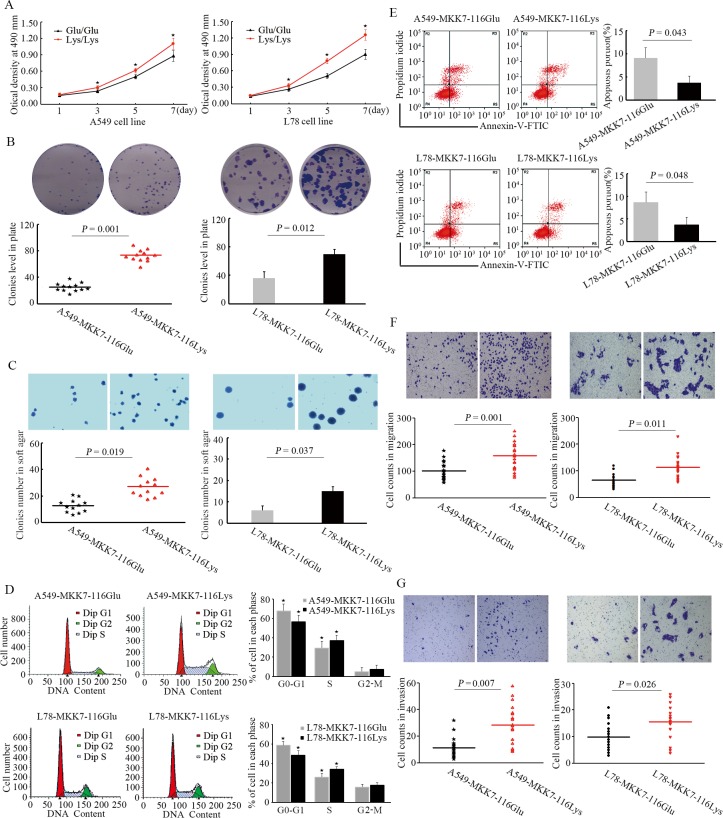
Effects of *MKK7* p.Glu116Lys on cellular proliferation, apoptosis, migration, and invasion. (A). A549 and L78 cells were seeded into 96-well plates after transfected with *MKK7*-116Glu or *MKK7*-116Lys lentivirus, and cell proliferation was evaluated every other day for a week using the MTT assay. OD values from four independent experiments were assessed (* indicated that a statistical significance with *P* < 0.05 between the groups). (B). Representative colony formation assay in 6-well plates both for A549 and L78 cells. The comparison of different colonies level between *MKK7*-116Glu-cells and *MKK7*-116Lys-cells was assessed by student’s *t*-test. (C). In soft agar assay, colonies number in cells with over-expressing *MKK7*-116Lys were much higher than cells transfected with *MKK7*-116Glu. Colonies were stained with crystal violet, and were counted in four randomly selected points in each well under the microscopy (original magnification: ×100). (D). Cell cycle analysis of A549 and L78 cells after transfection with lentiviruses containing different p.Glu116Lys allele. (E). Annexin V-FITC/PI apoptosis assay of A549 and L78 cells after transfection with *MKK7*-116Glu or *MKK7*-116Lys lentivirus. (F, G). Cell migration and invasion assays were performed. The upper chambers were seeded with various cell lines. The membranes of the chambers were stained with crystal violet. All data were representative of at least three separate experiments.

To explore the effects of the *MKK7* p.Glu116Lys rare variant on cell biological behaviors, multitudinously functional experiments were further executed. The proliferation test showed that cells with over-expressing *MKK7*-116Lys displayed a higher proliferation potential than cells with over-expressing *MKK7*-116Glu (**Fig 2A**, ANOVA test *P*<0.001). Cells highly expressing *MKK7*-116Lys also had strikingly promoted abilities of colony formation in common plate, as well as in soft-agar, compared to the cells with M*KK7*-116Glu (**Fig 2B** and 2**C**). In addition, we further performed flow cytometry to evaluate the influence of p.Glu116Lys variants on cells cycle and apoptosis. We found that the over-expressing *MKK7*-116Lys in A549 cells induced a significantly reduction in the G0/G1 phase (12.9% decreased, *P* = 0.015) and a corresponding increase in the G2/M phase (7.4% increased, *P* = 0.038), while compared with the cells stably expressing *MKK7*-116Glu (**Fig 2D**). Notably, the A549 cells with *MKK7*-116Lys also had decreased apoptosis rate than cells with *MKK7*-116Glu (**Fig 2E**, *P* = 0.043). Furthermore, cells with highly expression of *MKK7*-116Lys showed remarkably promoted migration and invasion capabilities in comparison to cells with over-expressing *MKK7*-116Glu (**Fig 2F** and 2**G**). These arresting results also occurred in the L78 cells with stably over-expressing *MKK7*-116Lys. All these findings suggested that the *MKK7*-116Lys variant had a detrimental impact on promoting cell proliferation, invasion and immigration.

### Effects of *MKK7* p.Glu116Lys on tumor growth and metastasis

**Fig 3:**
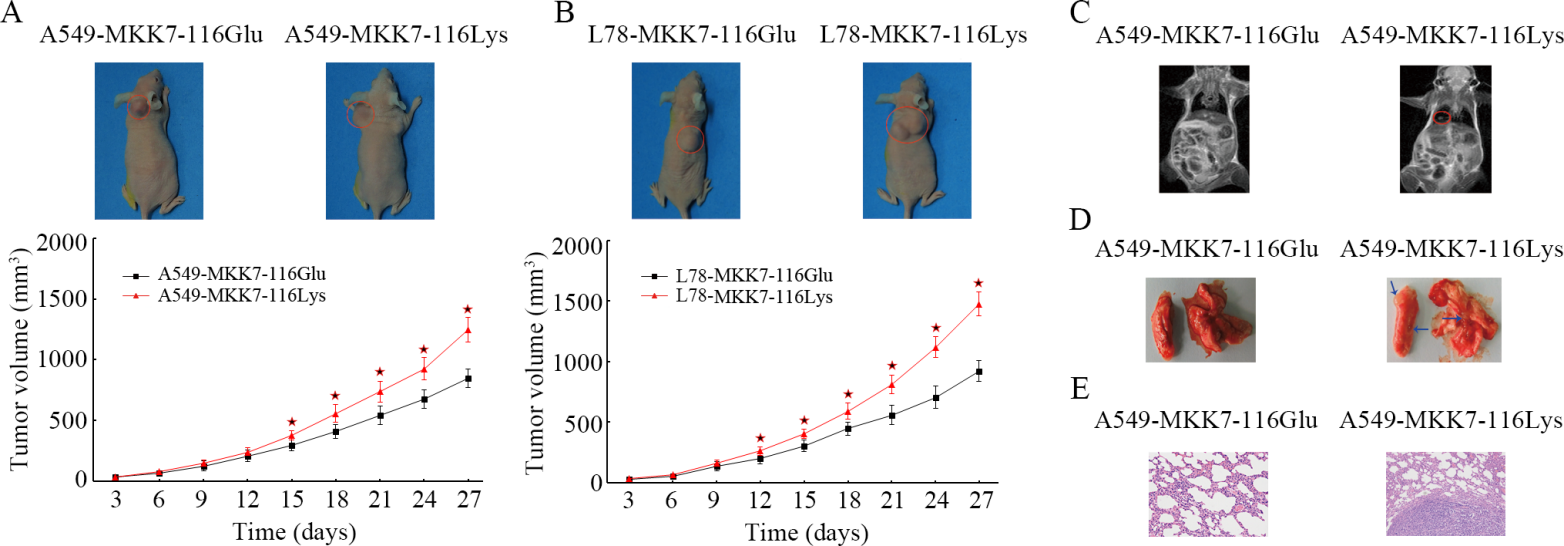
The effects of *MKK7* p.Glu116Lys on tumor growth and metastasis. (A, B). Subcutaneously implanted *MKK7*-116Glu (A549*-MKK7*-116Glu and L78*-MKK7*-116Glu cells) and *MKK7*-116Lys (A549*-MKK7*-116Lys and L78*-MKK7*-116Lys cells) cells xenografted tumors were established and were observed for a total of 4 weeks. Tumor volumes represented the mean ± SD of 6 mice per group. Columns, mean; bars, SD. The symbol “^★^” indicated a statistical significance with *P* < 0.05 between the cells transfected with the two different transfectants. (C-E). A549*-MKK7*-116Glu or A549*-MKK7*-116Lys cells were separately injected into the tail vein of each mouse. After proximately 10 weeks, lung metastases were evaluated using magnetic resonance imaging, macroscopic observation and histomorphology under microscopy. The red loops and arrows indicate the metastases.

To further determine the effect of p.Glu116Lys on tumor growth and metastasis *in vivo*, cells with stably over-expressing *MKK7*-116Glu or *MKK7*-116Lys were injected into nude mice subcutaneously (both for A549 and L78 cell lines), and intravenously (for A549 cell line only), respectively. As is shown in **Fig 3A**, the injection of *MKK7*-116Lys cells resulted in tumor formation began 4 days earlier compared to the results from injection of *MKK7*-116Glu cells. The tumor grew faster, and after 4 weeks, the tumor size in the former group was larger than the latter group (For A549: 1246.3±102.3 mm^3^ *vs*. 846.3±78.5 mm^3^, *P* <0.001, **Fig 3A**; for L78: 1474.5±99.4 mm^3^ *vs*. 921.1±88.4 mm^3^, *P* <0.001, **Fig 3B**). Moreover, we used the MRI and histology examination to determine whether the *MKK7* p.Glu116Lys could cause tumor metastases, and found that all the mice injected with A549 cells over-expressing *MKK7*-116Lys suffered from pulmonary metastasis, while the mice group injected with *MKK7*-116Glu A549 cells did not (**Fig 3C**, **3D** and **3E**). These findings demonstrated that *MKK7*-116Lys variant enhanced lung tumor growth and metastasis *in vivo*.

### Effects of *MKK7* with p.Glu116Lys rare variant on the gene expression profiles

To decipher the potential mechanisms behind the *MKK7* with p.Glu116Lys rare variant induced lung cancer risk and progression, we further performed DGE sequencing to compare gene profiles between A549-*MKK7*-116Glu cells and A549-*MKK7*-116Lys cells. We found that compared to cells with stably over-expressing *MKK7*-116Glu, cells with *MKK7*-116Lys had 192 genes differentially expression with a q value of <0.001 (**S1 File**). Among these genes, 128 genes were up-expressed, and 64 genes were down-expressed. We further validated the DGE results using qRT-PCR assay, with detecting differently expressed genes including 5 up-expression genes(*STC2*, *SLC1A3*, *MSMO1*, *BCL10* and *HMGCR*) and 5 down-expression genes(*SAA1*, *SBK2*, *CDH5*, *COL4A2* and *BCL9L*). The results were in concordance with those findings through DGE sequencing. Furthermore, we conducted Gene Ontology (GO) analysis using these differentially expressed genes. The GO results indicated that these 192 differentially expression genes were annotated to be associated with cell cycle process, cell proliferation, apoptosis, tissue development, tumor invasion, and metastasis et al. Above results suggests that alteration from 116Glu to Lys in *MKK7* might influence its downstream targets expression and thus facilitate lung cancer initiation and development.

## Discussion

In the current study conducted among southern and eastern Chinese with a total of 5,016 lung cancer patients and 5,181 controls, we estimated the relationships between rare variants in *MKK7* gene and lung cancer risk and prognosis, and found that the p.Glu116Lys rare variant was significantly associated with an increased lung cancer risk, progression and prognosis. The individuals with 116Lys variants had promotional cancer risk and higher probability of metastasis at diagnosis. The harmful role of the 116Lys variants also resulted in a poorer lung cancer prognosis in the patients than in the patients with Glu/Glu genotype. Further functional assays demonstrated that lung cancer cells with p.116Lys variant accelerated cell growth, proliferation, colony formation, migration and invasion. They also promoted the xenograft growth and metastasis of nude mice *in vivo* through regulating a serial of cancer-related genes. However, no conspicuous evidence was obtained to prove any significant association between other rare SNPs and lung cancer risk and prognosis. To the best of our knowledge, this is the first study to investigate the associations between the genetic rare variants in *MKK7* and lung cancer risk, as well as metastasis and prognosis.

Accumulating evidence indicated that ‘missing heritability’ in complex human diseases caused increasing attention over the past a few years because the findings from the GWAS and other epidemiological studies did not completely explained the genetic heritability [4, 5, 24]. With rapid advances in high-throughput sequencing technologies, unnoticed genetic components such as low-frequency (1%≤MAF< 5%) and rare genetic variants (MAF< 1%) are being thoroughly assessed and investigated for their associations with complex human diseases [6, 7, 25]. These approaches highlight an unparalleled opportunity to decipher unexplained genetic contributions in forming complex traits [26, 27], especially in human malignancies [10].

*MKK7* has been identified to be a tumor suppressor gene constitutive activation of *JNK* signaling pathway to induce cell apoptosis [28, 29]. Recently, a study reported that tissue-specific inactivation of the stress signaling kinase *MKK7* in *ras*-driven lung carcinomas and *NeuT*-driven mammary tumors markedly accelerates tumor onset and reduces overall survival through directly coupling oncogenic and genotoxic stress to the *p53* stability[11]. Lin HJ et al. identified that *MKK7* could negatively regulate the expressions of *MMP-2* and *MMP-9* and thus inhibited cancer cell migration and invasion [17]. In addition, another report showed that ectopic expression of the *MKK7* suppresses the formation of overt metastases by inhibiting the ability of disseminated cells to colonize the lung[14]. Furthermore, several studies display an intimate linkage with germline mutations in *MKK7* and cancer onset and progression [30–32]. In the present study, we found that p.Glu116Lys rare variant in *MKK7* contributed a pernicious impact on lung cancer risk and prognosis. We also observed a remarkable interaction between clinical stage and the rare variant on cancer survival. The p.Glu116Lys variant located at kinase activity domain of the *MKK7* gene, which might influence the structure and functions of the *MKK7* based on the bioinformatics analysis (http://snpinfo.niehs.nih.gov/). Our biological assays demonstrated that the 116 locus alteration from Glu to Lys in *MKK7* could promote cells proliferation, migration, invasion, and reduce cells apoptosis *in vitro*; the adverse role of 116Lys variant was also found to facilitate the xenograft growth and metastasis *in vivo*. The 116Lys variant further altered the expression of downstream genes modulated by *MKK7* as the DGE results showed, which might be closely related with lung cancer initiation and development. Among these differentially expressed genes, there were ones annotated as cell-regulated genes, cell apoptosis, cancer-related genes, tumor invasion and metastasis. For example, as the DGE results had indicated, *STC2*, *YEATS4* and *SLC1A3* genes were up-expressed in the cells with stably over-expressing *MKK7*-116Lys compared with the cells with *MKK7*-116Glu. A study had reported higher mRNA and protein expressions of *STC2* in lung cancer tissues compared to the adjacent normal tissue. Knockdown of *STC2* slowed down lung cancer cell growth progression, colony formation and metastasis [33]. Another article showed the proof that overexpression of *YEATS4* abrogated senescence in human bronchial epithelial cells, while RNAi-mediated attenuation of *YEATS4* could conversely reduce lung cancer cells proliferation and tumor growth, impair colony formation, and induce cellular senescence[34]. In addition, several genes such as *CDH5* and *UBA7* (also known as *UBE1L*) were significantly down-regulated in the cells with *MKK7*-116Lys. A previous study had reported a downregulation of *CDH5* in Bulgarian patients with early-stage non-small cell lung cancer [35]. Loss of *UBE1L* is a common event in lung carcinogenesis, and the *UBE1L* gene suppressed lung cancer growth by preferentially inhibiting *cyclin D1* [36, 37]. All the above public evidence was in accordance with our findings in the current study, which convincingly supported our ultimatums that *MKK7* p.Glu116Lys rare variant exerted adverse effects on lung cancer risk, progression and prognosis by modulating a number of cancer-related genes.

Our study has several strengths and limitations. Based on three independent case-control studies, we have obtained consistent results of the association between the *MKK7* p.Glu116Lys rare variant and lung cancer risk and prognosis, with a compellingly strong study power of 100% (two-sided test, α = 0.05) to detect an OR of 3.29 for the 116Lys variant genotypes (which occurred at a frequency of 2.7% in the controls), and with a 100% statistical power for HR with a value to 1.53, while compared with the 116Glu wild-genotype. A serial of functional experiments further sustained the results that the p.116Lys variants conferred noxious effects on lung cancer risk, progression and prognosis. However, there are also some limitations. The selection bias is unavoidable on account of the hospital-based retrospective studies. Also, with restriction to a Chinese Han population, it is uncertain whether our findings could be generalized to other populations. Furthermore, due to the technological limitations, we did not promulgate any direct target genes of *MKK7* with respect to the p.Glu116Lys rare polymorphism, which might help us to understand the precisely molecular mechanism of this rare SNP on influencing cancer risk and progression.

In conclusion, our findings indicated that the p.Glu116Lys rare variant of *MKK7* was associated with an increased lung cancer risk and worsened prognosis in Chinese, which was likely to be related to modulation of a serial of cancer-related genes. These results suggested that the *MKK7* p.Glu116Lys may be a useful predictive biomarker for lung cancer susceptibility and prognosis. Validations through larger population-based studies in different ethnic groups, and functional assay to reveal target gene of the p.Glu116Lys rare SNP in *MKK7* are warranted.

## Materials and Methods

### Ethics statement

Each participant was scheduled for an interview to collect individual information on smoking status, alcohol use, and other selected factors, and to obtain a donated 5 mL of peripheral venous blood under his or her informed consent. The study was approved by the institutional review boards of Guangzhou Medical University (Ethics Committee of Guangzhou Medical University: GZMC2007-07-0676) and Soochow University (Ethics Committee of Soochow University: SZUM2008031233). All experiments and procedures involving animals were conducted in accordance with guidelines approved by the Laboratory Animal Center of Guangzhou Medical University.

### Study population and follow-up

In this study, three two-stage independently retrospective studies with a total of 5,015 lung cancer patients and 5,181 healthy controls were performed in southern and eastern Chinese populations. In brief, 1,559 lung cancer cases and 1,679 cancer-free controls as the discovery set, which included southern Chinese with 1,056 primary lung cancer cases and 1,056 healthy subjects recruited from the Guangzhou city, and eastern Chinese with 503 patients and 623 controls enrolled from Suzhou city, have been previously described[1, 38, 39]. In the validation set I, 1,949 lung cancer patients that were continuously recruited from Guangzhou between April 2009 and June 2014 with a 90% response rate and 1,957 sex and age (± 5 years) frequency matched cancer-free controls who were randomly selected from about 3,000 individuals participating in health community programs with an 83% response rate were used. Moreover, the other population from Suzhou city was used as validation set II, in which 1,508 lung cancer cases were enrolled between December 2009 and March 2014 with an 85% response rate and 1,545 controls were randomly selected from 8000 participators in the annual healthy checkup programs with a response rate of 91%. All the participants were genetically unrelated ethnic Han Chinese and none had blood transfusion in the last six months. Definitions of smoking status, pack-years smoked, drink status, family history of cancer and family history of lung cancer have been previously described[38, 39].

As was previously reported, clinical information and characteristics of patients were also collected [40]. Patient follow-ups were performed through telephone calls every three months from time of enrollment to the last scheduled follow-up or death. Survival time was calculated starting from the day the patients first received confirmed diagnoses to the date of the last follow-up or death, and dates of death were acquired from medical records or information provided by family members through telephone follow-ups. Patients that were lost to follow-ups or had no accurate data on clinical information were excluded. In the finalized study, 908 patients from the discovery set, 1027 patients from validation set I and 971 patients from validation set II that have completed the follow-up and had intact survival data were included in this study. In addition, to eliminate the bias in patient selection, we analyzed the differences in clinical features, as well as in survival data, between the included and excluded groups, and no deviated results were observed.

### SNP selection and genotype determination

Because no published data reveal potentially functional variants in *MKK7*, we only selected those exon variants in gene coding region causing amino acid change that are supposed to be with most functional potential. Through the strategy of searching for the rare polymorphisms located in the *MKK7* gene exons region based on the public dbSNP database (http://www.ncbi.nlm.nih.gov/snp/, access to 1/1/2014), we found that 5 SNPs of *MKK7* gene (i.e., rs28395770G>A: p.Glu116Lys, rs56316660A>G: p.Asn118Ser, rs56106612C>T: p.Arg138Cys, rs55800262G>A: p.Ala195Thr and rs1053566C>T: p.Leu259Phe) were rare with MAF<1% in Chinese population. We then re-sequenced the whole cDNA of *MKK7* in 100 normal Chinese Hans randomly picked from the controls, and no newfound rare variants outside of those 5 SNPs were obtained. Therefore, we chose these above rare SNPs in the current study.

Genomic DNA was extracted from 2 mL peripheral blood using the routine method. Genotypes of all the selected SNPs were determined by direct DNA sequencing. A fragment of a total of 1,102 bp from the whole genomic DNA templates with the forward primer 5′-CCCAGCATTGAGATTGACCAGA-3′ and reverse primer 5′- TGCCATGTAGGCGGCACA-3′, which comprises the 5 studied SNPs was amplified. The PCR program for the amplification was as follows: 95°C for 5 minutes and then 40 cycles of denaturation (95°C for 45 seconds), annealing (61°C for 1 minute), and extension (72°C for 1 minute and 30 seconds), and a final polymerization step at 72°C for 7 minutes. The products were then separated by a 1% agarose gel and extracted. Finally, the PCR products were sequenced by an automated sequencing system (ABI Prism 3730 Genetic Analyzer; Applied Biosystems, Foster City, USA) operating according to the manufactures’ protocols (**S1 Fig**).

### Plasmids construction, lentivirus package and cell transfection

The cDNA sequence of human *MKK7* gene with a wild-type (p.116Glu) was synthesized by the Sangon Biotech Company (Shanghai, China) and cloned into pLVX-IRES-neo expression vector (Clontech Laboratories Inc., San Francisco, CA, USA). The mutated pLV-*MKK7*-116Lys plasmid was induced by site-directed mutagenesis using the Quick Change XL site-directed mutagenesis kit (Stratagene, La Jolla, CA, USA). The resulting constructs were verified by direct sequencing. The lentiviral production and transduction were performed abiding by protocol described elsewhere [40]. In brief, replication-defective VSV-G pseudotyped viral particles were packaged using a 3-plasmid transient cotransfection method (Lenti-T HT packaging mix, Clonetech, San Francisco, CA, USA). Viruses were then harvested and concentrated. For transfection, two human lung cancer cell lines, A549 (a human lung adenocarcinoma cell line) and L78 (a human lung squamous carcinoma cell line) were infected with control lentivirus (an “empty” vector without the *MKK7* fragment inserted), pLV-*MKK7*-116Glu lentivirus and the pLV-*MKK7*-116Lys lentivirus, respectively. The cells were stably selected with G418 at 100 μg/ml (Gibco, Lyon, France), and the drug-resistant cells were confirmed by qRT-PCR and western blotting assays (**S2 Fig**).

### Cell viability assay

Cells infected with different allele lentivirus (pLV-*MKK7*-116Glu and pLV-*MKK7*-116 Lys) were seeded into 96-well flat-bottomed plates. 1,000 cells per 100 μl of cell suspension were used to add in each well. After a certain time of cultivation, cell viability was measured by MTT assay as is previously described [40]. In brief, 20 μl MTT solutions (5 mg/mL, Sigma, USA) per well were added for 4 h before the end of the experiment. After that, the supernatant fluid was removed and 150 μl of DMSO was added to each well. The absorbance was then measured at 490 nm wavelength using a Plate Reader (Bio-Tec Instruments, Inc.) after shaking the plate for 15 min at room temperature.

### Flow cytometry analysis of cell cycle and apoptosis

For cell cycle analysis, cells with stably expressing *MKK7*-116Glu or *MKK7*-116Lys were collected, washed with PBS and fixed by 70% ethanol for at least 1 h. Subsequently, the cells were stained with 0.5 mL propidium iodide (PI) staining solution, and cellular DNA content was analyzed using a flow cytometry (BD Biosciences, CA, USA). For cell apoptosis, an annexin v-fluoresce-in isothiocyanate (V-FITC)/PI double staining assay was conducted according to the manufacturer instructions. In brief, the cells were harvested and stained with annexin V-FITC and PI for 20 min at room temperature in the dark. The cells were then washed twice with PBS, and the fluorescence of the cells was measured by flow cytometry.

### Colony formation assay

Cells with stably over-expressing *MKK7*-116Glu or *MKK7*-116Lys were seeded into a 6-well plate (100 cell/well) with RPMI 1640 medium supplemented with 10% fetal bovine serum (FBS), and allowed to grow until visible colonies formed (approximately 2 weeks). After washing with PBS, the cell colonies were fixed with 4% paraformaldehyde and stained with crystal violet (Invitrogen) for 30 min, then washed, air dried, photographed and counted. Furthermore, colony formation assay in soft-agar was also executed to detect the effect of *MKK7* Glu116Lys rare variant on cell malignant transformation. The detailed procedures were previously described [40]. Briefly, cells suspended with DMEM medium containing a concentration of 0.35% soft agar were poured onto 6-cm tissue culture dishes coated with 5 ml of 0.75% bottom agar. At the end of the experiment, the colonies were then stained, photographed and counted.

### Transwell migration assay and matrigel invasion assay

Cell migration and invasion abilities were appraised by Corning transwell insert chambers (8-uM pore size; Costar, USA) and BD BioCoat Matrigel Invasion Chamber (Becton Dickinson Biosciences, USA), respectively. 2×10^4^ (migration assay) or 2×10^5^ (invasion assay) transfected cells in 200μl serum-free RPMI 1640 medium were seeded in the upper chamber, and 800 μl medium with 10% FBS were added to the lower compartment. After 24 h for migration assays or 48h for invasion assays at 37°C in a 5% CO_2_ humidified atmosphere, cells in the upper chamber were carefully scraped off using a cotton swab, and the cells that had migrated to or invaded the lower surfaces of the membrane were fixed with 4% paraformaldehyde solution and stained with crystal violet (Invitrogen), imaged and counted. Assays were independently conducted for three times.

### Xenografts in mice

Female BALB/c nude mice that were 4–5 weeks of age were purchased from the Laboratory Animal Center of Guangdong province (Guangzhou, China). Cells with *MKK7*-116Glu or *MKK7*-116Lys were diluted to a concentration of 5×10^7^/ml in physiological saline. 0.1 ml of the cells suspension was injected subcutaneously into the dorsal flank of mice to construct tumor growth model (both for A549 and L78 cell lines), or injected intravenously into the caudal vein of mice to construct tumor metastasis model (for A549 cell line only). Six nude mice were used for each group. When a tumor was palpable in the growth model, tumor size was measured every other day using a caliper along two perpendicular axes and calculated according to the following formula: Volume = 1/2×length×width^2^. The tumor metastases were evaluated by magnetic resonance imaging (MRI) and histology examination.

### Magnetic resonance imaging

MRI was performed proximately 10 weeks post-injection using Philips Gyroscan Intera 1.5T ultraconducted MRI scanner (Netherlands) and incorporating a removable gradient coil insert. The details of MRI imaging were conducted as suggested by the public literature [41]. In brief, mice were placed prone on an MR-compatible sled within a carrier tube and positioned in the magnet. Induction and maintenance of anesthesia during imaging was achieved through inhalation of 10% chloral hydrate. MRI examination of coronal T2-weighted (T2WI) scanning was conducted with the following variables: Repetition time (TR) = 4000ms, echo time (TE) = 111ms, field-of-view (FOV) = 3 cm, number of slices = 20, slice thickness = 1.0 mm, matrix = 256×256. Following image acquisition, raw image sets were transferred to a processing workstation and processed using the medical imaging software. Tumor metastatic burden were calculated from manually traced regions-of-interest (ROI).

### Histological analysis

The animals were euthanized and their tumor masses were harvested and fixed with 10% neutral formalin solution, embedded in paraffin, and sectioned at 5 μm. The sections were then stained with hematoxylin-eosin (HE) staining and examined by light microscopy at 20× magnification.

### RNA extraction and genes profiling sequencing

The total RNAs from different A549 transfectant cells were extracted using the TRIzol reagent (Invitrogen) in accordance with the manufacturer instructions. RNA quantity and quality were assessed using a NanoDrop 2000 spectrophotometer (Thermo Scientific, MA, USA). The gene expression profiling both in A549-*MKK7*-116Glu cells and A549-*MKK7*-116Lys cells were conducted using Illumina NlaIII digital gene expression (DGE) sequencing. Analyses were performed according to the manufacturer recommendations [42]. Briefly, DGE sequence libraries were sequenced using Illumina HiSeq 2000 platform. Differentially expressed genes between the two groups of cells were identified using the reads per kilobase of transcript per million mapped reads (RPKM) method. The q value ≤ 0.001 and the absolute value of log2 ratio ≥ 1 were as the threshold to judge the significance of gene expression differences.

### Quantitative real-time PCR analysis

On the basis of genes profiling sequencing results, the expression levels of 10 selected genes (includes 5 up-expression genes and 5 down-expression genes) in the A549-*MKK7*-116Glu cells and A549-*MKK7*-116Lys cells were verified by the quantitative real time PCR (qRT-PCR) assay described elsewhere [39]. The relative levels of RNA were detected using the ABI Prism 7900HT sequence detection system (Applied Biosystems) and with the SYBRPremix Ex Taq (Perfect Real Time, TaKaRa, China) and *β-actin* as the internal reference. Each assay was performed in triplicate and independently repeated three times. All the primers used for PCR amplification are listed in **S5 Table**.

### Statistical analysis

The chi-square test was used to assess differences in the distributions of demographic characteristics between cases and controls. The distributions of genotypes between cases and controls were analyzed with Fisher’s exact test. Unconditional logistic regression model with or without adjustment for surrounding factors was used to evaluate the associations between the *MKK7* rare SNPs and lung cancer risk and metastasis. The correlations between *MKK7* rare genotypes and lung cancer clinical features were tested using Spearman rank correlation. The sequence kernel association test (SKAT) was used to estimate the combined effect of multiple variants in *MKK7* and lung cancer risk using R software (version 3.0.2; The R Foundation for Statistical Computing) with the SKAT package[43]. The REML model was used to assess the heritability explained by the genetic variants [44]. Breslow-Day test was used to test the homogeneity between the subgroups. The statistical power was calculated using the PS Software. The false-positive report probability (FPRP) test was applied to detect false-positive association findings [45]. The associations between clinical variables, as well as genotypes, and overall survival time were estimated using the Kaplan-Meier method and Log-rank test. The Cox proportional hazards regression model with or without adjustment for confounders was used to evaluate the effect of rare polymorphisms on lung cancer prognosis. Multiplicative interactions were assessed by logistic regression or Cox regression [38]. The differences in gene expression, colonies number levels, and cells’ ability to invade and migrate were analyzed using the student’s *t*-test. Repeated measure ANOVA test was performed to analyze the deviation of cell proliferation and tumor growth in different groups. All tests were two-sided using the SAS software (version 9. 3; SAS Institute) and *P* <0.05 was considered statistically significant.

## Supporting Information

S1 Fig Genotyping of rare SNPs in *MKK7* by direct DNA sequencing. (TIF)

S2 Fig *MKK7* expression levels in A549 and L78 cells transfected with the *MKK7*-116Glu vector, the *MKK7*-116Lys vector and an “empty” vector. (A). *MKK7* mRNA expression in A549 cells transfected with different transfectants. (B). *MKK7* mRNA expression in L78 cells transfected with different transfectants. (C). *MKK7* protein levels in A549 cells transfected with different transfectants. (D). *MKK7* protein levels in L78 cells transfected with different transfectants.
(TIF)

S1 Table Associations between *MKK7* p.Glu116Lys genotypes and lung cancer progression (DOC)

S2 Table Associations between *MKK7* rare polymorphisms’ assembly and the risk of lung cancer. (DOC)

S3 Table Analysis of the effects of patients’ demographic and clinical characteristics on lung cancer survival. (DOC)

S4 Table Associations between *MKK7* rare SNP’ assembly and lung cancer survival in the total populations. (DOC)

S5 Table Sequence of primers used in real time RT-PCR analysis. (DOC)

S1 File Differentially expressed genes between A549-*MKK7*-116Lys cells and A549-*MKK7*-116Glu cells. (XLS)
