# Supplementary figures and images for: The MKK7 p.Glu116Lys Rare Variant Serves as a Predictor for Lung Cancer Risk and Prognosis in Chinese
Source: PLoS Genet. 2016 Mar 30;12(3):e1005955. doi: 10.1371/journal.pgen.1005955 (PMC4814107; doi:10.1371/journal.pgen.1005955)

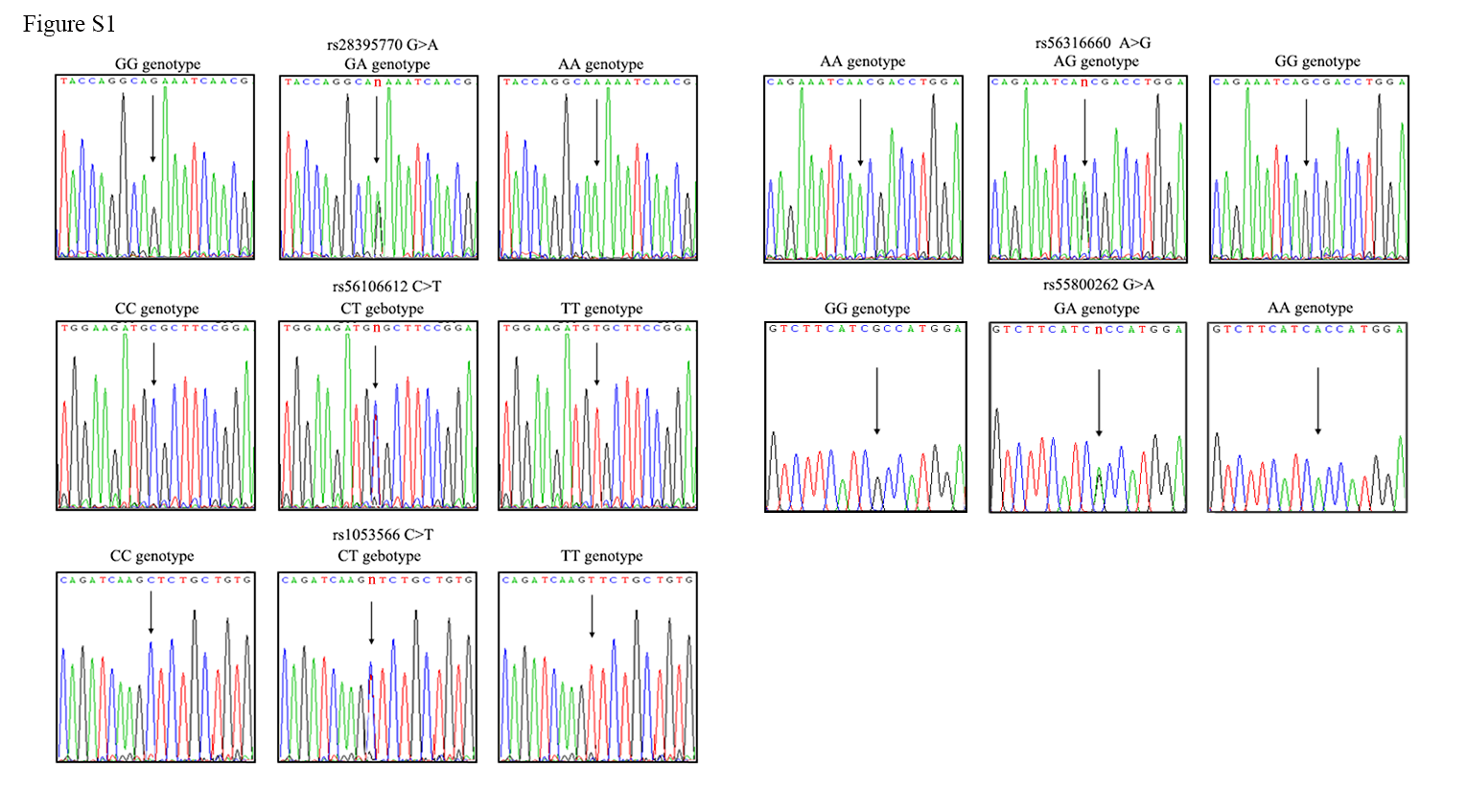

Supplement: S1 Fig — (TIF) [file pgen.1005955.s001.tif]

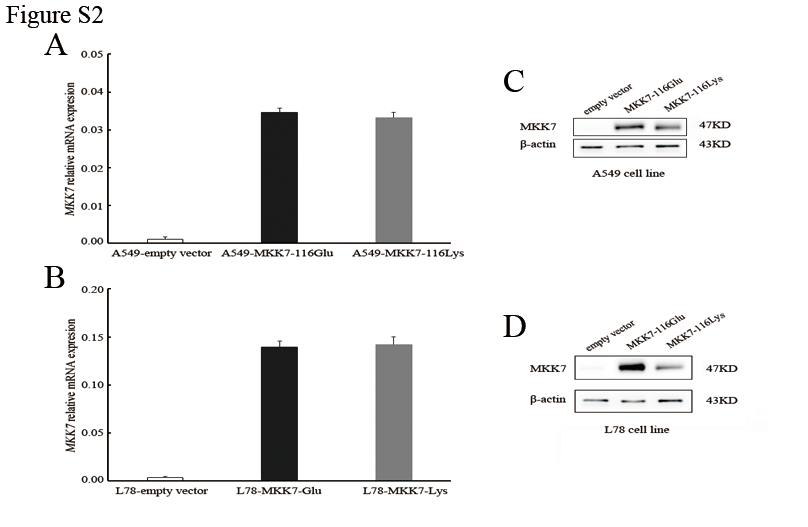

Supplement: S2 Fig — (A). MKK7 mRNA expression in A549 cells transfected with different transfectants. (B). MKK7 mRNA expression in L78 cells transfected with different transfectants. (C). MKK7 protein levels in A549 cells transfected with different transfectants. (D). MKK7 protein levels in L78 cells transfected with different transfectants. (TIF) [file pgen.1005955.s002.tif]
