# Supplementary material for: The MKK7 p.Glu116Lys Rare Variant Serves as a Predictor for Lung Cancer Risk and Prognosis in Chinese
Source: PLoS Genet. 2016 Mar 30;12(3):e1005955. doi: 10.1371/journal.pgen.1005955 (PMC4814107; doi:10.1371/journal.pgen.1005955)
Supplement: S1 Table — (DOC) [file pgen.1005955.s003.doc]

**S1_Table**. Associations between *MKK7* p.Glu116Lys genotypes and lung cancer progression

| Clinical stages | p.Glu116Lys | |  | Adjusted OR (95% CI) ***^a^*** | Correlation analysis *P*-value *^b^* |
| --- | --- | --- | --- | --- | --- |
|  | Glu/Glu | Lys/Glu+Lys/Lys |  | Lys/Lys +Lys/Glu *vs.* Glu/Glu |  |
| Stages |  |  |  |  | **<0.001** |
| I+II | 1061(93.9) | 69(6.1) |  | 1.00(ref.) |  |
| III | 1430(92.3) | 120(7.7) |  | 1.30(0.95-1.76) |  |
| IV | 2105(90.1) | 231(9.9) |  | **1.69(1.28-2.24)** |  |
|  |  |  |  |  |  |
| Nodal metastasis |  |  |  |  | **<0.001** |
| 0 | 1178(94.2) | 73(5.8) |  | 1.00(ref.) |  |
| 1 | 1014(91.5) | 94(8.5) |  | **1.51(1.10-2.07)** |  |
| 2 | 1480(91.3) | 141(8.7) |  | **1.53(1.14-2.06)** |  |
| 3 | 924(89.2) | 112(10.8) |  | **1.95(1.43-2.65)** |  |
|  |  |  |  |  |  |
| Distal metastasis |  |  |  |  | **0.003** |
| 0 | 2491(93.0) | 189(7.0) |  | 1.00(ref.) |  |
| 1 | 2105(90.1) | 231(9.9) |  | **1.45(1.18-1.77)** |  |
|  |  |  |  |  |  |
| Metastasis |  |  |  |  | **0.002** |
| No | 831(94.9) | 45(5.1) |  | 1.00(ref.) |  |
| Yes | 3765(90.9) | 375(9.1) |  | **1.84(1.34-2.53)** |  |

*^a^* Adjusted in logistic regression models that included age, sex, smoking status, drinking status, and family history of cancer.

*^b^* *P* value of spearman correlation analysis between *MKK7* Glu116Lys genotypes and selected clinical feature.
