# Supplementary material for: The MKK7 p.Glu116Lys Rare Variant Serves as a Predictor for Lung Cancer Risk and Prognosis in Chinese
Source: PLoS Genet. 2016 Mar 30;12(3):e1005955. doi: 10.1371/journal.pgen.1005955 (PMC4814107; doi:10.1371/journal.pgen.1005955)
Supplement: S2 Table — (DOC) [file pgen.1005955.s004.doc]

**S2_Table.** Associations between *MKK7* rare polymorphisms’ assembly and the risk of lung cancer.

| *MKK7* rare polymorphisms’ assembly: |  | Case(n=5016)  n(%) | Control(n=5181)  n(%) | Adjusted OR  (95% CI)***^a^*** | *P* value***^b^*** | AIC value***^c^*** |  | SKAT *P* value |  | Fisher test *P* value |
| --- | --- | --- | --- | --- | --- | --- | --- | --- | --- | --- |
| p.Glu116Lys +p.Asn118Ser +p.Arg138Cys  +p.Ala195Thr + p.Leu259Phe *^d^* |  |  |  |  |  |  |  |  |  |  |
| SNPs(1,0,0,0,0) |  |  |  |  |  |  |  |  |  |  |
| Yes |  | 381(7.6) | 128(2.5) | **3.24(2.64-3.97)** | **4.06×10^-29^** | **13840.2** |  | **3.22×10^-36^** |  | **1.18×10^-33^** |
| No |  | 4635(92.4) | 5053(97.5) |  |  |  |  |  |  |  |
| SNPs(0,1,0,0,0) |  |  |  |  |  |  |  |  |  |  |
| Yes |  | 47(0.9) | 42(0.8) | 1.18(0.77-1.80) | 0.442 | 13983.5 |  | 0.069 |  | 0.524 |
| No |  | 4969(99.1) | 5139(99.2) |  |  |  |  |  |  |  |
| SNPs(0,0,1,0,0) |  |  |  |  |  |  |  |  |  |  |
| Yes |  | 45(0.9) | 32(0.6) | 1.37(0.87-2.17) | 0.177 | 13982.3 |  | 0.101 |  | 0.110 |
| No |  | 4971(99.1) | 5149(99.4) |  |  |  |  |  |  |  |
| SNPs(0,0,0,1,0) |  |  |  |  |  |  |  |  |  |  |
| Yes |  | 47(0.9) | 36(0.7) | 1.37(0.88-2.13) | 0.159 | 13982.1 |  | 0.078 |  | 0.187 |
| No |  | 4969(99.1) | 5145(99.3) |  |  |  |  |  |  |  |
| SNPs(0,0,0,0,1) |  |  |  |  |  |  |  |  |  |  |
| Yes |  | 34(0.7) | 27(0.5) | 1.38(0.83-2.29) | 0.222 | 13982.6 |  | 0.107 |  | 0.369 |
| No |  | 4982(99.3) | 5154(99.5) |  |  |  |  |  |  |  |
| SNPs(1,1,0,0,0) |  |  |  |  |  |  |  |  |  |  |
| Yes |  | 13(0.3) | 4(0.1) | **3.16(1.02-9.76)** | **0.045** | 13979.4 |  | **5.60×10^-39^** |  | **0.028** |
| No |  | 5003(99.7) | 5177(99.9) |  |  |  |  |  |  |  |
| SNPs(1,0,1,0,0) |  |  |  |  |  |  |  |  |  |  |
| Yes |  | 8(0.2) | 3(0.1) | 3.07(0.81-11.7) | 0.100 | 13981.1 |  | **3.60×10^-39^** |  | 0.140 |
| No |  | 5008(99.8) | 5178(99.9) |  |  |  |  |  |  |  |
| SNPs(1,0,0,1,0) |  |  |  |  |  |  |  |  |  |  |
| Yes |  | 9(0.2) | 3(0.1) | 2.99(0.81-11.1) | 0.102 | 13981.0 |  | **4.47×10^-39^** |  | 0.087 |
| No |  | 5007(99.8) | 5178(99.9) |  |  |  |  |  |  |  |
| SNPs(1,0,0,0,1) |  |  |  |  |  |  |  |  |  |  |
| Yes |  | 4(0.1) | 1(0.1) | 4.70(0.52-42.6) | 0.169 | 13981.7 |  | **1.25×10^-38^** |  | 0.211 |
| No |  | 5012(99.9) | 5180(99.9) |  |  |  |  |  |  |  |
| SNPs(0,1,1,0,0) |  |  |  |  |  |  |  |  |  |  |
| Yes |  | 3(0.1) | 1(0.1) | 2.75(0.28-26.6) | 0.383 | 13983.2 |  | 0.098 |  | 0.367 |
| No |  | 5013(99.9) | 5180(99.9) |  |  |  |  |  |  |  |
| SNPs(0,1,0,1,0) |  |  |  |  |  |  |  |  |  |  |
| Yes |  | - | - | - | - | - |  | - |  | - |
| No |  | - | - | - |  |  |  |  |  |  |
| SNPs(0,1,0,0,1) |  |  |  |  |  |  |  |  |  |  |
| Yes |  | 3(0.1) | 1(0.1) | 3.97(0.41-38.3) | 0.234 | 13982.4 |  | 0.150 |  | 0.367 |
| No |  | 5013(99.9) | 5180(99.9) |  |  |  |  |  |  |  |
| SNPs(0,0,1,1,0) |  |  |  |  |  |  |  |  |  |  |
| Yes |  | 0(0.0) | 2(0.1) | - | 0.953 | 13981.5 |  | 0.127 |  | 0.500 |
| No |  | 5016(100.0) | 5179(99.9) |  |  |  |  |  |  |  |
| SNPs(0,0,1,0,1) |  |  |  |  |  |  |  |  |  |  |
| Yes |  | 2(0.1) | 2(0.1) | 0.99(0.14-7.11) | 0.989 | 13984.1 |  | 0.237 |  | 1.00 |
| No |  | 5014(99.9) | 5179(99.9) |  |  |  |  |  |  |  |
| SNPs(0,0,0,1,1) |  |  |  |  |  |  |  |  |  |  |
| Yes |  | 2(0.1) | 0(0.0) | - | 0.955 | 13982.0 |  | 0.150 |  | 0.242 |
| No |  | 5014(99.9) | 5181(100.0) |  |  |  |  |  |  |  |
| SNPs(1,1,1,0,0) |  |  |  |  |  |  |  |  |  |  |
| Yes |  | - | - | - | - | - |  | - |  | - |
| No |  | - | - | - |  |  |  |  |  |  |
| SNPs(1,1,0,1,0) |  |  |  |  |  |  |  |  |  |  |
| Yes |  | 4(0.1) | 1(0.1) | 4.52(0.50-40.9) | 0.179 | 13981.8 |  | **6.99×10^-41^** |  | 0.211 |
| No |  | 5012(99.9) | 5181(99.9) |  |  |  |  |  |  |  |
| SNPs(1,1,0,0,1) |  |  |  |  |  |  |  |  |  |  |
| Yes |  | - | - | - | - | - |  | - |  | - |
| No |  | - | - |  |  |  |  |  |  |  |
| SNPs(1,0,1,1,0) |  |  |  |  |  |  |  |  |  |  |
| Yes |  | - | - | - | - | - |  | - |  | - |
| No |  | - | - |  |  |  |  |  |  |  |
| SNPs(1,0,1,0,1) |  |  |  |  |  |  |  |  |  |  |
| Yes |  | 1(0.1) | 0(0.0) | - | 0.950 | 13982.7 |  | **4.76×10^-41^** |  | 0.492 |
| No |  | 5015(99.9) | 5181(100.0) |  |  |  |  |  |  |  |
| SNPs(1,0,0,1,1) |  |  |  |  |  |  |  |  |  |  |
| Yes |  | - | - | - | - | - |  | - |  | - |
| No |  | - | - |  |  |  |  |  |  |  |
| SNPs(0,1,1,1,0) |  |  |  |  |  |  |  |  |  |  |
| Yes |  | 0(0.0) | 1(0.1) | - | 0.951 | 13982.8 |  | 0.215 |  | 0.508 |
| No |  | 5016(100.0) | 5180(99.9) |  |  |  |  |  |  |  |
| SNPs(0,1,1,0,1) |  |  |  |  |  |  |  |  |  |  |
| Yes |  | - | - | - | - | - |  | - |  | - |
| No |  | - | - |  |  |  |  |  |  |  |
| SNPs(0,1,0,1,1) |  |  |  |  |  |  |  |  |  |  |
| Yes |  | 0(0.0) | 2(0.1) | - | 0.955 | 13982.2 |  | 0.126 |  | 0.500 |
| No |  | 5016(100.0) | 5179(99.9) |  |  |  |  |  |  |  |
| SNPs(0,0,1,1,1) |  |  |  |  |  |  |  |  |  |  |
| Yes |  | - | - | - | - | - |  | - |  | - |
| No |  | - | - |  |  |  |  |  |  |  |
| SNPs(1,1,1,1,0) |  |  |  |  |  |  |  |  |  |  |
| Yes |  | - | - | - | - | - |  | - |  | - |
| No |  | - | - |  |  |  |  |  |  |  |
| SNPs(1,1,1,0,1) |  |  |  |  |  |  |  |  |  |  |
| Yes |  | - | - | - | - | - |  | - |  | - |
| No |  | - | - |  |  |  |  |  |  |  |
| SNPs(1,1,0,1,1) |  |  |  |  |  |  |  |  |  |  |
| Yes |  | - | - | - | - | - |  | - |  | - |
| No |  | - | - |  |  |  |  |  |  |  |
| SNPs(1,0,1,1,1) |  |  |  |  |  |  |  |  |  |  |
| Yes |  | - | - | - | - | - |  | - |  | - |
| No |  | - | - |  |  |  |  |  |  |  |
| SNPs(0,1,1,1,1) |  |  |  |  |  |  |  |  |  |  |
| Yes |  | - | - | - | - | - |  | - |  | - |
| No |  | - | - |  |  |  |  |  |  |  |
| SNPs(1,1,1,1,1) |  |  |  |  |  |  |  |  |  |  |
| Yes |  | - | - | - |  | - |  | - |  | - |
| No |  | - | - |  |  |  |  |  |  |  |

***^a^*** Data were calculated by logistic regression analysis adjusted with surrounding factors. ***^b^*** *P* value were calculated by multivariate logistic regression analysis.

***^c^*** AIC value calculated in multivariate logistic regression model. *^d^* In the genotypes assembly of these 5 SNPs, p.Glu116Lys was placed in the first position, p.Asn118Ser in the second, p.Arg138Cys in the third, p.Ala195Thr in the forth, and p.Leu259Phe in the last. The wild-genotype of the 5 SNPs was coded as 0, and their heterozygous or homozygous variant genotype was coded as 1. For example, the Yes of SNPs(1,0,0,0,0) indicated that individual was with 116Lys variants(Lys/Glu or Lys/Lys) and the other SNPs were all wild-genotype; the No of SNPs(1,0,0,0,0) indicated that individual was with wild-genotype for all 5 SNPs.
