# Supplementary material for: The MKK7 p.Glu116Lys Rare Variant Serves as a Predictor for Lung Cancer Risk and Prognosis in Chinese
Source: PLoS Genet. 2016 Mar 30;12(3):e1005955. doi: 10.1371/journal.pgen.1005955 (PMC4814107; doi:10.1371/journal.pgen.1005955)
Supplement: S3 Table — (DOC) [file pgen.1005955.s005.doc]

**S3_Table.** Analysis of the effects of patients’ demographic and clinical characteristics on lung cancer survival

| Variables | Discovery set | |  | Validation set I | |  | Validation set II | |  | MST  (months) | Log-rank  *P* value | HR (95%CI) ^a^ |
| --- | --- | --- | --- | --- | --- | --- | --- | --- | --- | --- | --- | --- |
|  | cases  n (%) | No. of  death |  | cases  n (%) | No. of  death |  | cases  n (%) | No. of  death |  |  |  |  |
| Total | 908 | 841 |  | 1027 | 767 |  | 971 | 672 |  |  |  |  |
| Age (years) |  |  |  |  |  |  |  |  |  |  | **9.21×10^-8^** |  |
| ≤ 60 | 430(47.4) | 394 |  | 522(50.8) | 367 |  | 518(53.4) | 355 |  | 16 |  | 1.00 (ref.) |
| > 60 | 478(52.6) | 447 |  | 505(49.2) | 400 |  | 453(46.6) | 317 |  | 12 |  | **1.24(1.15-1.35)** |
| Sex |  |  |  |  |  |  |  |  |  |  | **0.019** |  |
| Male | 649(71.5) | 605 |  | 730(71.1) | 549 |  | 691(71.2) | 476 |  | 13 |  | 1.00 (ref.) |
| Female | 259(27.9) | 236 |  | 297(28.9) | 218 |  | 280(28.8) | 196 |  | 16 |  | **0.90(0.82-0.99)** |
| Family history of cancer |  |  |  |  |  |  |  |  |  |  | 0.070 |  |
| No | 837(92.2) | 775 |  | 946(92.1) | 715 |  | 890(91.7) | 619 |  | 14 |  | 1.00 (ref.) |
| Yes | 71(7.8) | 66 |  | 81(7.9) | 51 |  | 81(8.3) | 53 |  | 15 |  | 0.87(0.74-1.02) |
| Family history of  lung cancer |  |  |  |  |  |  |  |  |  |  | 0.083 |  |
| No | 877(96.6) | 813 |  | 998(97.2) | 751 |  | 943(97.1) | 654 |  | 14 |  | 1.00 (ref.) |
| Yes | 31(3.4) | 28 |  | 29(2.8) | 15 |  | 28(2.9) | 18 |  | 21 |  | 0.80(0.62-1.05) |
| Smoking status |  |  |  |  |  |  |  |  |  |  | **6.69×10^-4^** |  |
| Never | 418(46.0) | 384 |  | 493(48.0) | 363 |  | 371(38.2) | 253 |  | 15 |  | 1.00 (ref.) |
| Ever | 490(54.0) | 457 |  | 534(52.0) | 404 |  | 600(61.8) | 419 |  | 13 |  | **1.15(1.06-1.25)** |
| Drinking status |  |  |  |  |  |  |  |  |  |  | 0.174 |  |
| Never | 751(82.7) | 692 |  | 821(79.9) | 624 |  | 793(81.7) | 537 |  | 14 |  | 1.00 (ref.) |
| Ever | 157(17.3) | 149 |  | 206(20.1) | 143 |  | 178(18.3) | 135 |  | 13 |  | 1.08(0.97-1.19) |
| Surgery |  |  |  |  |  |  |  |  |  |  | **3.79×10^-7^** |  |
| No | 539(59.4) | 511 |  | 624(60.8) | 474 |  | 634(65.3) | 442 |  | 13 |  | 1.00 (ref.) |
| Yes | 369(40.6) | 330 |  | 403(39.2) | 293 |  | 337(34.7) | 230 |  | 16 |  | **0.81(0.74-0.88)** |
| Chemotherapy |  |  |  |  |  |  |  |  |  |  | **1.74×10^-8^** |  |
| No | 334(36.8) | 318 |  | 333(32.4) | 258 |  | 355(36.6) | 277 |  | 11 |  | 1.00 (ref.) |
| Yes | 574(63.2) | 523 |  | 694(67.6) | 509 |  | 616(63.4) | 395 |  | 16 |  | **0.79(0.72-0.86)** |
| Radiotherapy |  |  |  |  |  |  |  |  |  |  | **0.002** |  |
| No | 398(43.8) | 365 |  | 586(57.1) | 427 |  | 515(53.0) | 344 |  | 12 |  | 1.00 (ref.) |
| Yes | 510(56.2) | 476 |  | 441(42.9) | 340 |  | 456(47.0) | 328 |  | 16 |  | **0.88(0.81-0.96)** |
| Stages |  |  |  |  |  |  |  |  |  |  | **1.83×10^-6^** |  |
| I+II | 164(18.0) | 134 |  | 219(21.4) | 138 |  | 223(23.0) | 151 |  | 17 |  | 1.00 (ref.) |
| III | 302(33.3) | 284 |  | 326(31.7) | 238 |  | 310(31.9) | 231 |  | 14 |  | **1.31(1.17-1.48)** |
| IV | 442(48.7) | 423 |  | 482(46.9) | 390 |  | 438(45.1) | 290 |  | 13 |  | **1.32(1.17-1.46)** |
| Histological types |  |  |  |  |  |  |  |  |  |  | **0.021** |  |
| Adenocarcinoma | 356(39.2) | 327 |  | 462(45.0) | 343 |  | 533(54.9) | 346 |  | 14 |  | 1.00 (ref.) |
| Squamous cell carcinoma | 289(31.8) | 267 |  | 314(30.6) | 248 |  | 286(29.5) | 205 |  | 15 |  | 1.06(0.96-1.16) |
| Large cell carcinoma | 44(4.9) | 40 |  | 40(3.9) | 26 |  | 24(2.5) | 22 |  | 13 |  | 1.04(0.84-1.29) |
| Small cell lung cancer | 113(12.4) | 108 |  | 134(13.0) | 101 |  | 94(9.7) | 77 |  | 12 |  | 1.22(1.07-1.39) |
| Other *^b^* | 106(11.7) | 99 |  | 77(7.5) | 49 |  | 34(3.5) | 22 |  | 15 |  | 0.93(0.78-1.09) |

Abbreviations: MST, median survival time; HR, hazard ratio; ref., reference. Bold type: statistically significant, *P* < 0.05.

*^a^* Data was calculated by univariate cox regression analysis combined with discovery and validation sets.

*^b^* Mixed-cell or undifferentiated carcinoma.
