# Supplementary material for: The MKK7 p.Glu116Lys Rare Variant Serves as a Predictor for Lung Cancer Risk and Prognosis in Chinese
Source: PLoS Genet. 2016 Mar 30;12(3):e1005955. doi: 10.1371/journal.pgen.1005955 (PMC4814107; doi:10.1371/journal.pgen.1005955)
Supplement: S4 Table — (DOC) [file pgen.1005955.s006.doc]

**S4_Table.** Associations between *MKK7* rare SNP’ assembly and lung cancer survival in the total populations.

| *MKK7* rare polymorphisms’ assembly: | Patients. n(%) | Death | MST(months) | Log-rank *P* value | HR (95%CI)*^a^* |
| --- | --- | --- | --- | --- | --- |
| p.Glu116Lys +p.Asn118Ser +p.Arg138Cys +p.Ala195Thr + p.Leu259Phe *^b^* |  |  |  |  |  |
| SNPs(1,0,0,0,0) |  |  |  |  |  |
| No | 2695(92.7) | 2105 | 14 | **4.01×10^-8^** | 1.00 (ref.) |
| Yes | 211(7.3) | 174 | 9 |  | **1.58(1.35-1.85)** |
| SNPs(0,1,0,0,0) |  |  |  |  |  |
| No | 2879(99.1) | 2256 | 14 | 0.413 | 1.00 (ref.) |
| Yes | 27(0.9) | 23 | 12 |  | 1.28(0.85-1.94) |
| SNPs(0,0,1,0,0) |  |  |  |  |  |
| No | 2883(99.2) | 2258 | 14 | 0.112 | 1.00 (ref.) |
| Yes | 23(0.8) | 21 | 11 |  | 1.53(0.99-2.35) |
| SNPs(0,0,0,1,0) |  |  |  |  |  |
| No | 2880(99.1) | 2258 | 14 | 0.882 | 1.00 (ref.) |
| Yes | 26(0.9) | 21 | 10 |  | 1.11(0.72-1.70) |
| SNPs(0,0,0,0,1) |  |  |  |  |  |
| No | 2886(99.3) | 2262 | 14 | 0.144 | 1.00 (ref.) |
| Yes | 20(0.7) | 17 | 11 |  | 1.32(0.82-2.13) |
| SNPs(1,1,0,0,0) |  |  |  |  |  |
| No | 2893(99.5) | 2267 | 14 | **0.025** | 1.00 (ref.) |
| Yes | 13(0.5) | 12 | 8 |  | 1.74(0.99-3.08) |
| SNPs(1,0,1,0,0) |  |  |  |  |  |
| No | 2902(99.9) | 2277 | 14 | 0.299 | 1.00 (ref.) |
| Yes | 4(0.1) | 2 | 15.5 |  | 0.57(0.14-2.30) |
| SNPs(1,0,0,1,0) |  |  |  |  |  |
| No | 2900(99.8) | 2274 | 14 | 0.817 | 1.00 (ref.) |
| Yes | 6(0.2) | 5 | 24.5 |  | 1.11(0.46-2.68) |
| SNPs(1,0,0,0,1) |  |  |  |  |  |
| No | 2904(99.9) | 2277 | 14 | 0.473 | 1.00 (ref.) |
| Yes | 2(0.1) | 2 | 12 |  | 1.50(0.38-6.00) |
| SNPs(0,1,1,0,0) |  |  |  |  |  |
| No | 2904(99.9) | 2278 | 14 | 0.323 | 1.00 (ref.) |
| Yes | 2(0.1) | 1 | 8 |  | 0.47(0.07-3.35) |
| SNPs(0,1,0,1,0) |  |  |  |  |  |
| No | 2906(100.0) | 2279 | 14 | - | 1.00 (ref.) |
| Yes | - | - | - |  | - |
| SNPs(0,1,0,0,1) |  |  |  |  |  |
| No | 2904(99.9) | 2277 | 14 | 0.981 | 1.00 (ref.) |
| Yes | 2(0.1) | 2 | 21 |  | 1.36(0.34-5.45) |
| SNPs(0,0,1,1,0) |  |  |  |  |  |
| No | 2906(100.0) | 2279 | 14 | - | 1.00 (ref.) |
| Yes | - | - | - |  | - |
| SNPs(0,0,1,0,1) |  |  |  |  |  |
| No | 2906(100.0) | 2279 | 14 | - | 1.00 (ref.) |
| Yes | - | - | - |  | - |
| SNPs(0,0,0,1,1) |  |  |  |  |  |
| No | 2905(99.9) | 2278 | 14 | 0.912 | 1.00 (ref.) |
| Yes | 1(0.1) | 1 | 24 |  | 1.15(0.16-8.19) |
| SNPs(1,1,1,0,0) |  |  |  |  |  |
| No | 2906(100.0) | 2279 | 14 | - | 1.00 (ref.) |
| Yes | - | - | - |  | - |
| SNPs(1,1,0,1,0) |  |  |  |  |  |
| No | 2904(99.9) | 2278 | 14 | 0.359 | 1.00 (ref.) |
| Yes | 2(0.1) | 1 | 11 |  | 0.42(0.06-2.96) |
| SNPs(1,1,0,0,1) |  |  |  |  |  |
| No | 2906(100.0) | 2279 | 14 | - | 1.00 (ref.) |
| Yes | - | - | - |  | - |
| SNPs(1,0,1,1,0) |  |  |  |  |  |
| No | 2906(100.0) | 2279 | 14 | - | 1.00 (ref.) |
| Yes | - | - | - |  | - |
| SNPs(1,0,1,0,1) |  |  |  |  |  |
| No | 2906(100.0) | 2279 | 14 | - | 1.00 (ref.) |
| Yes | - | - | - |  | - |
| SNPs(1,0,0,1,1) |  |  |  |  |  |
| No | 2906(100.0) | 2279 | 14 | - | 1.00 (ref.) |
| Yes | - | - | - |  | - |
| SNPs(0,1,1,1,0) |  |  |  |  |  |
| No | 2906(100.0) | 2279 | 14 | - | 1.00 (ref.) |
| Yes | - | - | - |  | - |
| SNPs(0,1,1,0,1) |  |  |  |  |  |
| No | 2906(100.0) | 2279 | 14 | - | 1.00 (ref.) |
| Yes | - | - | - |  | - |
| SNPs(0,1,0,1,1) |  |  |  |  |  |
| No | 2906(100.0) | 2279 | 14 | - | 1.00 (ref.) |
| Yes | - | - | - |  | - |
| SNPs(0,0,1,1,1) |  |  |  |  |  |
| No | 2906(100.0) | 2279 | 14 | - | 1.00 (ref.) |
| Yes | - | - | - |  | - |
| SNPs(1,1,1,1,0) |  |  |  |  |  |
| No | 2906(100.0) | 2279 | 14 | - | 1.00 (ref.) |
| Yes | - | - | - |  | - |
| SNPs(1,1,1,0,1) |  |  |  |  |  |
| No | 2906(100.0) | 2279 | 14 | - | 1.00 (ref.) |
| Yes | - | - | - |  | - |
| SNPs(1,1,0,1,1) |  |  |  |  |  |
| No | 2906(100.0) | 2279 | 14 | - | 1.00 (ref.) |
| Yes | - | - | - |  | - |
| SNPs(1,0,1,1,1) |  |  |  |  |  |
| No | 2906(100.0) | 2279 | 14 | - | 1.00 (ref.) |
| Yes | - | - | - |  | - |
| SNPs(0,1,1,1,1) |  |  |  |  |  |
| No | 2906(100.0) | 2279 | 14 | - | 1.00 (ref.) |
| Yes | - | - | - |  | - |
| SNPs(1,1,1,1,1) |  |  |  |  |  |
| No | 2906(100.0) | 2279 | 14 | - | 1.00 (ref.) |
| Yes | - | - | - |  | - |

Abbreviations: MST, median survival time; HR, hazard ratio; ref., reference. Bold type: statistically significant, *P* < 0.05.

*^a^* The Cox regression analysis was adjusted for age, sex, smoking, stage, histology, surgery, chemo-therapy, and radio therapy status.

*^b^* In the genotypes assembly of these 5 SNPs, p.Glu116Lys was placed in the first position, p.Asn118Ser in the second, p.Arg138Cys in the third, p.Ala195Thr in the forth, and p.Leu259Phe in the last. The wild-genotype of the 5 SNPs was coded as 0, and their heterozygous or homozygous variant genotype was coded as 1. For example, the Yes of SNPs(1,0,0,0,0) indicated that individual was with 116Lys variants(Lys/Glu or Lys/Lys) and the other SNPs were all wild-genotype; the No of SNPs(1,0,0,0,0) indicated that individual was with wild-genotype for all 5 SNPs.
