# Supplementary material for: The MKK7 p.Glu116Lys Rare Variant Serves as a Predictor for Lung Cancer Risk and Prognosis in Chinese
Source: PLoS Genet. 2016 Mar 30;12(3):e1005955. doi: 10.1371/journal.pgen.1005955 (PMC4814107; doi:10.1371/journal.pgen.1005955)
Supplement: S5 Table — (DOC) [file pgen.1005955.s007.doc]

**S5_Table. Sequence of primers used in real time RT-PCR analysis.**

| **Symbol** |  | **Forward primer** |  | **Reverse primer** |
| --- | --- | --- | --- | --- |
| *MKK7* |  | CAACAGGACAGTTTCCCTACAA |  | CACCTCCAGCGTCTCGTAG |
| *STC2* |  | TGTGGCGTGTTTGAATGTTT |  | CACAGGTCGTGCTTGAGGTA |
| *SLC1A3* |  | CATGCACAGAGAAGGCAAAA |  | GTCACGGTGTACATGGCAAG |
| *MSMO1* |  | ATCCAGCTGCCTTTGATTTG |  | TTCCAAATGGAGCCTGAAAC |
| *BCL10* |  | AGGTCTGGACACCCTTGTTG |  | CAGTGGATGCCCTCAGTTTT |
| *HMGCR* |  | GTCATTCCAGCCAAGGTTGT |  | TCCTGTCCACAGGCAATGTA |
| *SAA1* |  | TGGTTTTCTGCTCCTTGGTC |  | CCCGAGCATGGAAGTATTTG |
| *SBK2* |  | CGAGCTCTACGAGGAAGTGC |  | CCGTCAGGAAGCTGTAGGAG |
| *CDH5* |  | GCCAGGTATGAGATCGTGGT |  | GTGTCTTCAGGCACGACAAA |
| *COL4A2* |  | AAGGAATCATGGGCTTTCCT |  | CTCTGGCACCTTTTGCTAGG |
| *BCL9L* |  | CCCAATCTCAGCACCAGAAT |  | CTCTTCACCTTGCCCTTGAG |
| *β-actin* |  | GGCGGCACCACCATGTACCCT |  | AGGGGCCGGACTCGTCATACT |
